# A multi-dimensional approach to unravel the intricacies of lactylation related signature for prognostic and therapeutic insight in colorectal cancer

**DOI:** 10.1186/s12967-024-04955-9

**Published:** 2024-02-28

**Authors:** Huixia Huang, Keji Chen, Yifei Zhu, Zijuan Hu, Yaxian Wang, Jiayu Chen, Yuxue Li, Dawei Li, Ping Wei

**Affiliations:** 1 Department of Oncology, Shanghai Medical College of Fudan University, Shanghai, China; 2 Department of Pathology, Fudan University Shanghai Cancer Center, Shanghai, China; 3 Cancer Institute, Fudan University Shanghai Cancer Center, Shanghai, China; 4 Institute of Pathology, Fudan University, Shanghai, China; 5 Department of Colorectal Surgery, Fudan University Shanghai Cancer Center, Shanghai, 200032 China; 6 Department of Oncology, Shanghai Medical College Fudan University, Shanghai, China

**Keywords:** Lactylation, Colorectal cancer, Tumor microenvironment, Immunotherapy, Tumor mutation burden, Drug sensitivity

## Abstract

**Background:**

Lactylation, a novel contributor to post-translational protein modifications, exhibits dysregulation across various tumors. Nevertheless, its intricate involvement in colorectal carcinoma, particularly for non-histone lactylation and its intersection with metabolism and immune evasion, remains enigmatic.

**Methods:**

Employing immunohistochemistry on tissue microarray with clinical information and immunofluorescence on colorectal cell lines, we investigated the presence of global lactylation and its association with development and progression in colorectal cancer as well as its functional location. Leveraging the AUCell algorithm alongside correlation analysis in single-cell RNA sequencing data, as well as cox-regression and lasso-regression analysis in TCGA dataset and confirmed in GEO dataset, we identified a 23-gene signature predicting colorectal cancer prognosis. Subsequently, we analyzed the associations between the lactylation related gene risk and clinical characteristics, mutation landscapes, biological functions, immune cell infiltration, immunotherapy responses, and drug sensitivity. Core genes were further explored for deep biological insights through bioinformatics and in vitro experiments.

**Results:**

Our study innovatively reveals a significant elevation of global lactylation in colorectal cancer, particularly in malignant tumors, confirming it as an independent prognostic factor for CRC. Through a comprehensive analysis integrating tumor tissue arrays, TCGA dataset, GEO dataset, combining in silico investigations and in vitro experiments, we identified a 23-gene Lactylation-Related Gene risk model capable of predicting the prognosis of colorectal cancer patients. Noteworthy variations were observed in clinical characteristics, biological functions, immune cell infiltration, immune checkpoint expression, immunotherapy responses and drug sensitivity among distinct risk groups.

**Conclusions:**

The Lactylation-Related Gene risk model exhibits significant potential for improving the management of colorectal cancer patients and enhancing therapeutic outcomes, particularly at the intersection of metabolism and immune evasion. This finding underscores the clinical relevance of global lactylation in CRC and lays the groundwork for mechanism investigation and targeted therapeutic strategies given the high lactate concentration in CRC.

**Supplementary Information:**

The online version contains supplementary material available at 10.1186/s12967-024-04955-9.

## Introduction

Colorectal cancer (CRC) ranks among the most commonly diagnosed cancers worldwide, stands as a formidable adversary in the realm of oncology, contributing significantly to global cancer-related morbidity and mortality [1, 2]. Research has conclusively demonstrated that early diagnosis and precise treatment of CRC can markedly enhance patient survival rate [3, 4]. Furthermore, commonly employed drugs for the treatment of CRC exhibit effectiveness in the initial phases of chemotherapy. However, it is noteworthy that certain drugs eventually develop resistance after multiple treatment cycles [5, 6]. In the realm of immune checkpoint inhibitor (ICI) which serves as a first-line treatment of MSI-H-dMMR metastatic CRC, their effectiveness is notably limited when it comes to immunologically "cold" tumors which are characterized by microsatellite stable (MSS) or mismatch repair proficient (pMMR) [7]. While remarkable breakthroughs have been realized through ICI therapy, either as a standalone approach or in combination with chemotherapy, there still exists a cohort of patients who struggle to find suitable treatment options due to the inherent heterogeneity and complexity of CRC. Notably, MSS-pMMR patients account for a large proportion of CRC patient [8]. Hence, to delve into the mechanism behind distinct treatment responses in contemporary CRC studies, researchers are actively exploring new biomarkers and pioneering approaches for early diagnosis and treatment. As researchers strive to improve the diagnosis, prevention, treatment and prognosis of this disease, innovative tools and novel approaches are emerging as promising allies in the battle against CRC.

Lactylation, a relatively recent addition to the realm of post-translational modifications (PTMs), involves the covalent attachment of lactate molecules to proteins, specifically through the chemical reaction between lactate (derived from lactic acid) and lysine residues on proteins [9]. This process, mediated by lactyltransferases [10, 11] and delactylases [10, 12, 13], is comparable to other PTMs like glycation and acetylation. Lactylation can exert its influence on a diverse range of proteins, both intracellular and extracellular. Histon lactylation, in particular, has been implicated in various processes such as macrophage polarization under hypoxic conditions [14], tumorigenesis [15], and other pathological process [16], while exploration of non-histon lactylation remains limited. Given that aerobic glycolysis is a hallmark of cancer [17], research on lactylation in cancer, especially CRC, is rapidly advancing to comprehend its biological and clinical significance. Colorectal cancer cells often exhibit metabolic traits, including lactate accumulation, impacting tumor initiation, development and immune environment [18–21]. Lactate accumulation and efflux in CRC cells are associated with epigenetic regulation of immune cells [18]. Targeting lactylation modifications, identified as a biological feature linked to colorectal cancer development and its related genes or pathways, may hold therapeutic potential for suppressing the metabolic and invasive capabilities of tumor cells, opening new avenue for immunotherapy and paving the way for innovative treatment strategies.

In this groundbreaking study, we have unveiled, for the first time, the intricate association between elevated lactylation levels and the development and progression of colorectal carcinoma, establishing it as an independent prognostic factor. Consequently, we devised a lactylation-related pattern that accurately reflects lactylation levels at the single-cell transcriptome level. This pattern underwent rigorous external validations, including in silico analyses and in vitro experiments, to elucidate lactylation characteristics in CRC. Our findings shed light on its role in predicting prognosis, providing mechanistic insights, and revealing potential treatment avenues in CRC. Given the notable concentration of lactate and heightened global lactylation levels in CRC, targeting lactylation and its related mechanisms emerges as a promising therapeutic strategy.

## Materials and methods

### Data acquisition

In this study, single-cell RNA sequencing (scRNA-seq) data for colorectal adenocarcinoma were obtained from the GSE132257 database (https://www.ncbi.nlm.nih.gov/geo/), comprising 10 CRC samples. The training cohort included RNA expression patterns of 589 patients with colorectal adenocarcinoma (COAD and READ) and corresponding clinical information from The Cancer Genome Atlas (TCGA) database (https://portal.gdc.cancer.gov/). Validation sets were derived from GSE39582 (579 patients) and GSE17536 (177 patients) expression profiles from the Gene Expression Omnibus (GEO) database (http://www.ncbi.nlm.nih.gov/geo). A total of 332 lactylation-related genes (LRGs) were selected based on a previous study [22] (Additional file 2: Table S1).

### scRNA-seq data processing and identification of lactylation-related genes (LRGs)

The scRNA-seq data were analyzed using the “Seurat” R program. Quality control involved filtering cells with a minimum expression in three cells, expressing 200–7000 genes in each cell, and no more than 20% mitochondrial genes. After normalization, 18,378 cells were identified. Data integration, identification of highly variable genes (HVGs), and principal component analysis (PCA) were performed. Cell clusters were established using the “FindClusters” and “FindNeighbors” functions, and cell annotation was conducted based on marker genes (Additional file 2: Table S2) and marker gene references (Additional file 2: Table S3, S4). Cell cycle heterogeneity was assessed, and lactylation activity scores were assigned using the “AUCell”. The “AUCell” R program, dedicated to assessing gene set activity, was employed to assign lactylation activity scores to individual cells. Subsequently, cells were categorized into high- and low-lactylation-AUC groups based on the median AUC score, and visualization was executed using the “ggplot2” R program. Differential analyses were then conducted to identify differentially expressed genes (DEGs) in high- and low-lactylation-AUC groups, resulting in the selection of 879 DEGs for further exploration (Additional file 2: Table S5). Additionally, correlation analysis was performed to identify genes strongly associated with lactylation activity, with the top 100 genes (Additional file 2: Table S6) in terms of association selected for future investigations which we termed CORGs. The DEGs and CORGs represented those with the most significant impact on lactylation activity (918 genes were obtained after intersection) (Additional file 2: Table S7).

### Functional enrichment analysis

To identify specific biological pathways enriched in high-lactylation cluster, we conducted a comprehensive analysis which included Gene Set Variation Analysis (GSVA) [23] (Additional file 2: Table S8), Gene Ontology (GO) analysis (Additional file 2: Table S9) and Disease Ontology (DO) analysis (Additional file 2: Table S10) to figure out the function of screened candidate genes and related pathways using the “ClustProfiler” and “GSVA” package. Kyoto Encyclopedia of Genes and Genomes (KEGG) analysis (Additional file 2: Table S12) was also performed to elucidate the function of selected core genes. Further, GSEA [24] was employed to identify enriched pathways or gene sets based on the differential expression results between high-risk and low-risk group. GSVA (Additional file 2: Table S13) and the correlation analysis between Hallmark pathways activities and LRG risk score were aimed to elucidate potential pathways associated with the identified signature.

### Construction and validation of LRGs risk signature

We performed univariate analysis on 918 genes associated with lactylation activity identified from scRNA-seq data to identify genes significantly correlated with patient survival (p < 0.05). Subsequently, a combination of least absolute shrinkage and selection operator (LASSO) and multivariate regression analysis was applied to further refine the gene selection and determine risk coefficients strongly associated with prognosis. The optimal regularization parameter (lambda) was determined through tenfold cross-validation, and genes with non-zero coefficients were considered as potential prognostic markers. The coefficients obtained from the multivariate analysis were used to calculate a risk score for each CRC patient (Additional file 2: Table S11). Patients from the TCGA-CRC dataset were stratified into high- and low-risk groups based on the Z-mean score, where a Z-mean score greater than 0 indicates the high-risk group, and less than 0 indicates the low-risk group. Kaplan–Meier survival curves were plotted, and log-rank tests were performed to assess the statistical significance of the observed differences in survival between the two risk groups. The predictive performance of the signature was evaluated using receiver operating characteristic (ROC) curves. The effectiveness of the prediction model was further validated in two independent GEO datasets through survival analysis and calculation of the area under the curve (AUC) in ROC analysis.

### Tumor mutation burden

The tumor mutation burden (TMB) was calculated from the somatic mutation profile of the TCGA cohort by “Maftools” package. The landscape of the LRG risk gene was plotted by waterfall chart. Substantially, the correlation between the mutation burden of the LRG risk gene and some top mutation genes was calculated and a spectrum of abnormal signaling pathways were highlighted.

### Immune cell infiltration analysis

In this study, the infiltration levels of immune cells in tumor microenvironment were estimated by 7 algorithms (“CIBERSORT”, “MCP counter”, “EPIC”, “ESTIMATE”, “TIMER”, “quanTIseq”, “IPS”) and was visualized by “ComplexHeatmap”. Specifically, we used the R package CIBERSORT [25] and single-sample gene set enrichment analysis (ssGSEA) algorithm [26] to determine the distribution of immune cell types and enrichment fraction of each immune cell between high- and low- risk groups. In addition, the expression levels of a few important immune checkpoint genes between two different risk groups were compared.

### Immunotherapy response prediction

The tumor immune dysfunction and exclusion (TIDE) algorithm [27] was initially employed to assess potential disparities in treatment responses between the high- and low-risk groups. A higher TIDE score was indicative of reduced efficacy, emphasizing a negative correlation between the TIDE score and treatment effectiveness. Subsequently, we utilized the SubMap tool [28] within the GenePattern software (http://www.broad.mit.edu/genepattern/) to analyze the difference between these two risk groups. In addition, we collected immunotherapy data from the GSE78220 dataset (melanoma) and the IMvigor210 dataset (urothelial carcinoma, UC). Within each dataset, we computed the LRG risk score to predict responses to immunotherapy.

### Nomogram construction

For better clinical practice, we assessed their independent prognostic values by univariable and multivariate Cox regression analysis to determine whether LRG risk score was an independent prognosis factor for predicting the survival of CRC patients. Besides, we compared the relationship between LRG risk and a series of clinical characteristics. To further enhance the prognostic accuracy and predictive ability of our model, we created a nomogram that used the risk score, T stage and N stage as independent prognostic criteria to compute the probability of OS at 1-, 3-, and 5- years. The calibration curve and decision curve analysis (DCA) were also utilized to evaluate the prediction discrimination and accuracy of the nomogram. The predictive efficacy was compared among different clinical pathological factors and the LRG risk score by AUC.

### Drug susceptibility prediction

We utilized the R package pRRophetic which incorporates a built-in ridge regression model to forecast drug response. Drug sensitivity data were achieved from the Cancer Therapeutics Response Portal (CTRP; https://portals.broadinstitute.org/ctrp) and Profiling Relative Inhibition Simultaneously in Mixtures (PRISM; https://depmap.org/portal/prism) databases. The area under the dose–response curve (AUC) values, reflecting drug sensitivity, were utilized, where lower AUC values indicate increased sensitivity to treatment response. To identify compounds with significantly lower estimated AUC values in the high-risk group (log2FC > 0.2 in CTRP and log2FC > 0.4 in PRISM), firstly, we conducted a differential drug response analysis. Subsequently, Spearman correlation analysis was performed to assess the relationship between AUC values and LRG scores. Our focus was on compounds displaying a negative correlation coefficient, specifically with Spearman's r values below − 0.30 for both CTRP and PRISM [29]. (Additional file 2: Table S14). Further analysis involved exploring the relationships between central LRG genes and chemotherapeutic drugs. We utilized the Genomics of Drug Sensitivity in Cancer (GDSC) database (https://www.cancerrxgene.org) to calculate half-maximal inhibition concentrations (IC50) for commonly used chemotherapeutic drugs [30]. Finally, we conducted Pearson correlation analysis to explore the correlations between LRGR core genes expression and drug sensitivity using datasets from Cancer Cell Line Encyclopedia (CCLE, https://sites.broadinstitute.org/ccle/) (Additional file 2: Table S15) and select the top correlated gene-drug pairs based on p-values. Visualization was conducted by ggplot2 package.

### Cell culture

Human CRC cell lines (HCT8, HCT15, HCT116, RKO, DLD1, SW480, SW620, SW1116) and human normal colon epithelial cell line NCM460 as well as murine CRC cell line MC38 were purchased from the American Type Culture Collection (ATCC). All cells were incubated at 37 °C with 5% CO2 and cultured in Dulbecco’s Modified Eagle Medium DMEM (BasalMedia, Shanghai, China) supplemented with 10% fetal bovine serum (FBS) and 1% penicillin/streptomycin, routinely tested for mycoplasma contamination.

### Immunofluorescence staining

On the preceding day, human- and murine-derived colorectal cancer cell lines including MC38, RKO, HCT15, HCT116, SW620 and DLD1 were planted in 4-well confocal dishes, with each well receiving a density of 1 × 10^5^ cells. The subsequent day, post-seeding, the cells underwent a rinse with phosphate-buffered saline (PBS), followed by fixation in 4% paraformaldehyde at room temperature for 20 min. After fixation, permeabilization was carried out using permeabilization buffer (Beyotime Biotech, Shanghai, China) for 20 min, succeeded by a 30-min blockage with immunostaining blocking buffer (Beyotime Biotech, Shanghai, China). Following this, the samples were subjected to an overnight incubation at 4 °C with the primary antibody pan-kla (#PTM-1401RM, PTMBio, Hangzhou, China) at a 1:100 dilution in blocking buffer. Subsequent to thorough washing, the cells were treated with fluorescent-labeled secondary antibodies for 1 h at room temperature. DAPI staining (Sigma, USA) was applied, and the observation was conducted using a confocal microscope (Olympus, Japan).

### Clinical specimen collection

We collected three groups of tissue samples, comprising 20 pairs of fresh frozen tissues stored in RNA later for RNA and protein extraction, obtained from patients between 2016 and 2017. Additionally, we obtained 10 pairs of formalin-fixed and paraffin-embedded tissue samples form patients between 2010 and 2018, along with a series of 10 × 16 tissue microarrays (TMA) with clinical information from CRC patients who underwent surgery at Fudan University Shanghai Cancer Center between January 2008 and September 2009. All samples included cases of CRC and adjacent normal tissues sourced from Fudan University Shanghai Cancer Center (FUSCC) without prior preoperative therapy. All clinicopathological diagnoses were confirmed by at least two pathologists according to the guidelines of the American Joint Committee on Cancer (AJCC). This research was conducted in strict accordance with the approval of the FUSCC Ethics Committee and written informed consent was obtained from each participating patient. Four pairs of tissues from group 1 were utilized for protein extraction to explore the heightened lactylation levels in tumor tissues, while the remaining 16 pairs were employed for RNA extraction to validate the mRNA expression of key genes. Immunohistochemistry (IHC) staining of AP2M1, TERF2IP, LY6E, ARL4C, ARPC1B were performed on the 10 pair of tissues from cohort 2. IHC of pan-kla and multiple immunohistochemistry staining of lactylation related genes were performed on TMA.

### Western blotting

Proteins were extracted from both cell lines and tissues using RIPA lysis buffer (Thermo Fisher Science, USA) in cold conditions. After centrifugation at 15000*g* for 10 min, the protein samples were subjected to immunoblotting. Protein concentration was determined using a BCA protein assay kit (Thermo Fisher Science, USA). Subsequently, the protein samples were loaded onto PAGE gels (Epizyme Biomedical Technology, Shanghai, China) and transferred to 0.22 µm Immobilon PVDF membranes (Millipore Sigma, USA). After blocking with 5% milk, the membranes were incubated in primary antibodies at appropriate dilutions overnight at 4 °C. Next, secondary antibodies (anti-Rabbit IgG) were applied at room temperature for approximately 1 h, and the immunoreactivity was visualized using an ECL system (Share-bio, Shanghai, China). The dilution factor for primary antibodies against pan-kla (#PTM-1401RM, PTMBio, Hangzhou, China) was 1:1000, and for Histone H3 (#P30266, Abmart, Shanghai, China) was 1:2000. All secondary antibodies were used at a dilution of 1:5000.

### RNA extraction and quantitative real-time PCR (qRT-PCR)

We isolated total RNA from CRC cell lines and patient samples using TRIzol reagent (Thermo Fisher Science, USA) with DNase treatment. Subsequently, reverse transcription was performed with ABScript II RT Mix (ABclonal, Wuhan, China) to synthesize cDNA which was subsequently stored at − 20 °C. Quantitative real-time PCR was conducted using TB Green Premix Ex Taq II (TaKaRa, Japan) and cDNA in triplicate on the ROCHE LightCycler®480 system. Fold changes in expression were achieved using the internal reference gene β-actin. Detailed primer sequences can be found in Additional file 2: Table S16.

### Immunohistochemical staining

In summary, paraffin-embedded tissue sections underwent a 2-h heat treatment at 65 °C, followed by a 30-min deparaffinization process involving three xylene treatments and subsequent 30-min rehydration using a graded ethanol series. Antigen retrieval was performed through a 20-min exposure to high heat and pressure in an EDTA buffer (pH 9.0). After blocking endogenous peroxidase activity and tissue blocking, sections were incubated with primary antibodies against AP2M1 (1:200, #ET1612-33, Proteintech, Wuhan, China), TERF2IP (1:50, #14595-1-AP; Proteintech, Wuhan, China), ARPC1B (1:500; #28368-1-AP; Proteintech, Wuhan, China), LY6E (1:200; #ab300399; abcam, England), ARL4C (1:200; #10202-1-AP; Proteintech, Wuhan, China) at room temperature for 1 h. Subsequently, biotin-conjugated secondary reagents were applied for 45 min. The IHC procedure was completed with DAB (3,3′-diaminobenzidine) (Genetech, China) staining and hematoxylin counterstaining. The final IHC scores, considering both staining percentage and intensity, were independently calculated by two experienced pathologists.

### Multiplex immunohistochemical staining

The deparaffinization, rehydration, and antigen retrieval process aligned with our previously described IHC protocol. Subsequently, we applied a pre-optimized antibody concentration and a predetermined staining sequence. Slides underwent incubation with both primary antibodies (TERF2IP, ARL4C, ARPC1B, CD8) and horseradish peroxidase-conjugated secondary antibodies, followed by tyramine signal amplification (TSA). Following each round of TSA, we executed antibody stripping and antigen retrieval procedures. Finally, DAPI (sigma, USA) was employed for nuclear staining.

### Statistical analysis

For normally distributed variables in two comparative groups, we applied unpaired Student’s t-tests to determine statistical significance. In instances where variables were non-normally distributed, Mann–Whitney U tests were employed for assessing the statistical significance between the two groups. When comparing more than two groups, we utilized parametric methods, specifically one-way ANOVA tests, and nonparametric methods, such as Kruskal–Wallis tests. To explore linear relationships, Pearson's or Spearman’s correlation analysis was conducted between pairs of variables. Kaplan–Meier survival analysis was performed to compare overall survival between high and low-risk groups, with the Log-rank test employed to assess differences in survival curves between the two groups. Furthermore, ROC curves were generated, and AUC values were calculated to evaluate the predictive performance of the risk score. All statistical analyses were carried out using R software (version 4.2.2) and GraphPad Prism 9. The data are presented as means ± SD, and statistical significance was set at p < 0.05. These rigorous analytical approaches ensure a robust evaluation of the study findings.

## Results

### Global lactylation level was up-regulated in CRC and associated with tumor progression

**Fig. 1:**
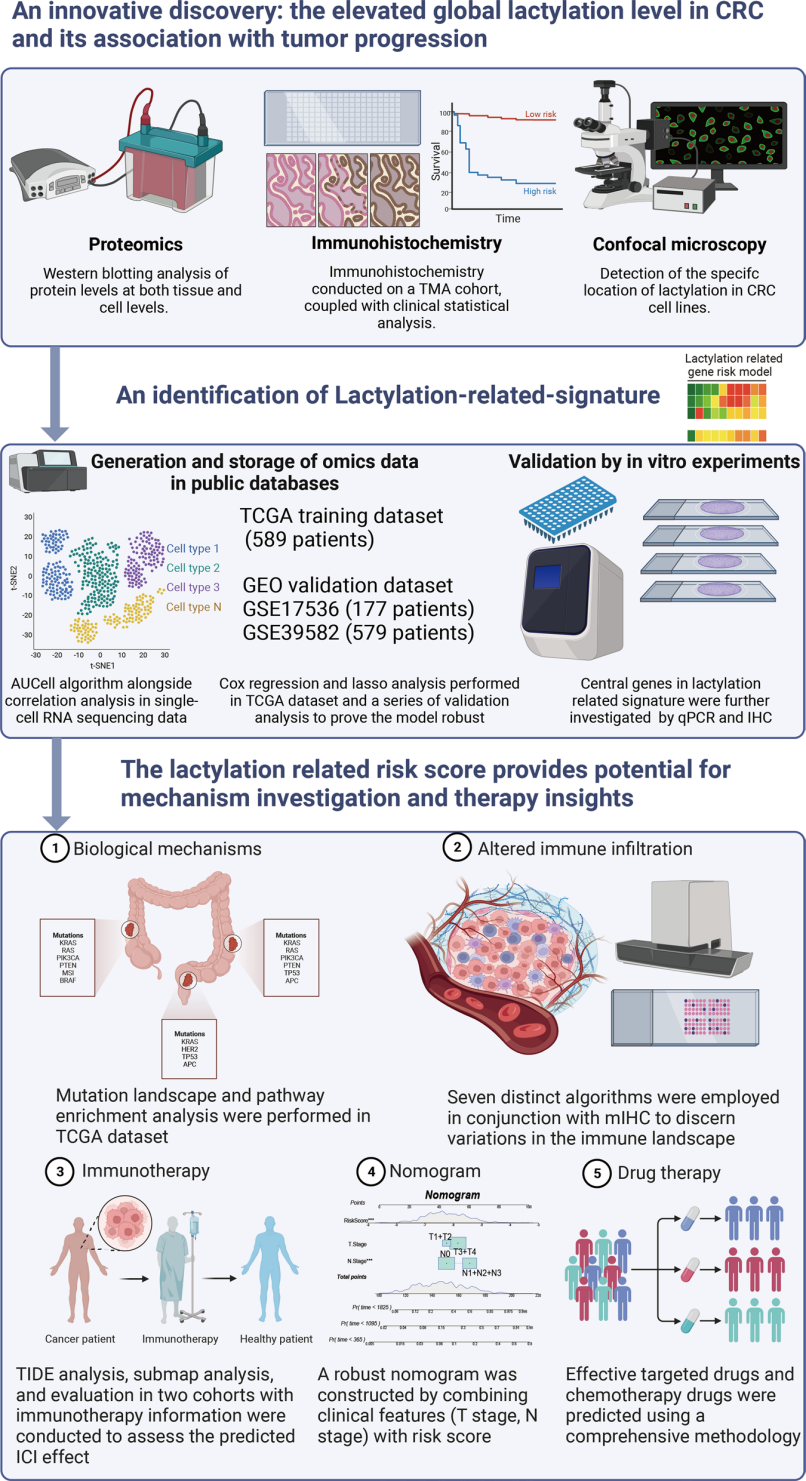
Workflow of the study

**Fig. 2:**
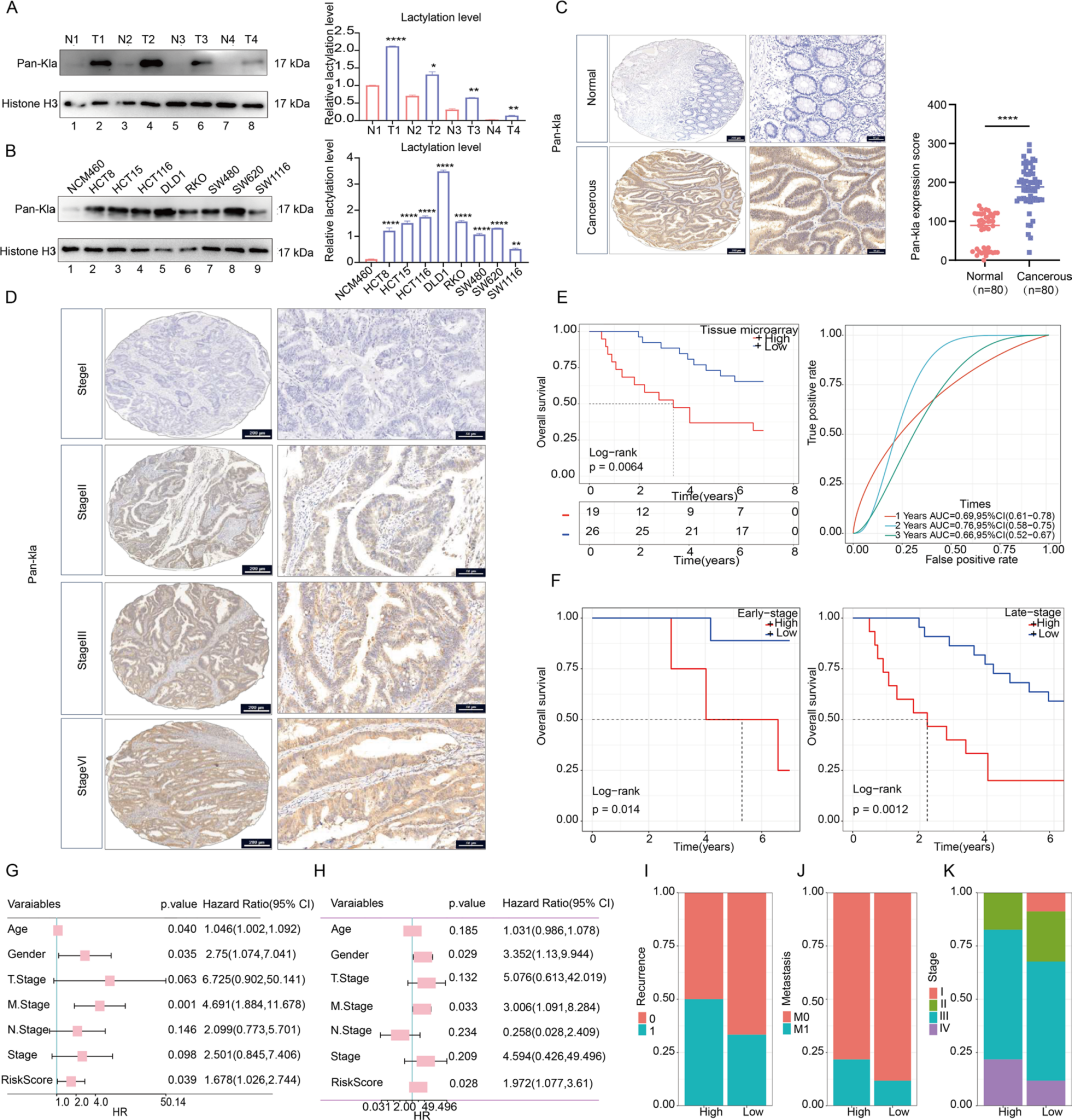
Global lactylation level was up-regulated in CRC and associated with tumor progression. **A** Western blotting analysis of lactylation levels in adjacent normal tissues (N) and cancerous tissues (T). **B** Assessment of lactylation levels in the colon epithelial cell line NCM460 and various CRC cell lines (HCT8, HCT15, HCT116, DLD1, RKO, SW480, SW620, SW1116) through western blotting. Densitometric analysis was conducted to quantify and statistically compare lactylation levels normalized to histone H3. **C**, **D** Immunohistochemical staining visualization of lactylation levels in normal and cancerous tissues (**C**) and the specific lactylation levels in tumor tissues at various stages from stage 1 to stage 4 (**D**) in a Tissue Microarray (TMA) cohort. A total of 80 normal colon tissues and 80 CRC tumors were analyzed. Statistical analysis was performed using the Mann–Whitney test. Scale bar: left panel, 200 μm; right panel, 50 μm. **E** Kaplan–Meier survival analysis assessing the relationship between lactylation level and OS of CRC patients, accompanied by a time-dependent ROC curve demonstrating the accuracy of the lactylation risk. **F** Kaplan–Meier survival analysis exploring the association between lactylation level and OS of CRC patients in early-stage (stages I–III) and late-stage (stage IV) patients. **G**, **H** Univariate and multivariate independent prognostic analysis conducted in the TMA cohort. **I**, **J** Distribution of recurrence (**I**), metastasis (**J**), and stage (**K**) in lactylation-high and lactylation-low patients. Statistical significance: *p < 0.05, **p < 0.01, ***p < 0.001, ****p < 0.0001

Previous researches indicated that CRC is notably characterized by heightened glycolytic activity [31]. Consequently, this metabolic trait leads to a significant increase in lactate production, a crucial substrate for histone lactylation. Due to the absence of a lactylation profile to confirm the presence of global lactylation in CRC, while in other cancers, lactylation has been demonstrated to exist in a variety of proteins and is crucial for the occurrence and development of tumor [15, 32], we are here to explore the existence of lactylation in CRC and its clinical significance (Fig. 1). Firstly, we conducted validation on our CRC samples, which revealed elevated global lactylation levels in cancerous tissues compared to adjacent normal tissues (Fig. 2A). This observation was further corroborated in CRC cell lines when compared to the normal colon epithelial cell line NCM46 (Fig. 2B). Additionally, we utilized immunofluorescence to assess the comprehensive lactylation status in CRC cell lines. The results indicated that lysine lactylation was not confined to the nucleus but was also present in the cytoplasm (Additional file 1: Fig. S1A–F). This observation further suggested that global lactylation extends beyond histone-related proteins and transcends the realm of transcriptional regulation, emphasizing its prevalence in diverse protein modifications. To assess the potential clinical significance of global lactylation, immunohistochemistry staining was performed on a comprehensive tissue microarray, unveiling a substantial increase in global lactylation levels in CRC tissues compared to normal tissues (Fig. 2C) and the level of lactylation rises as the stage increases (Fig. 2D). Moreover, our investigation demonstrated that higher global lactylation levels were associated with a poor prognosis, both in early-stage (stages I–III) and late-stage patients (stage IV) (Fig. 2E, F). Through univariate and multivariate Cox regression analyses, lactylation levels consistently emerged as an independent prognostic factor for CRC (Fig. 2G, H). Statistically, the high lactylation group exhibited a higher rate of recurrence and distant metastasis, coupled with more advanced tumor stages (Fig. 2I-K). These findings underscore the potential significance of lactylation as a prognostic marker in CRC, shedding light on its association with disease progression and outcomes. Further exploration of the molecular mechanisms underlying lactylation in CRC may unveil novel therapeutic avenues for this challenging malignancy.

### Identifying lactylation-related genes (LRGs) in CRC from single-cell transcriptome

**Fig. 3:**
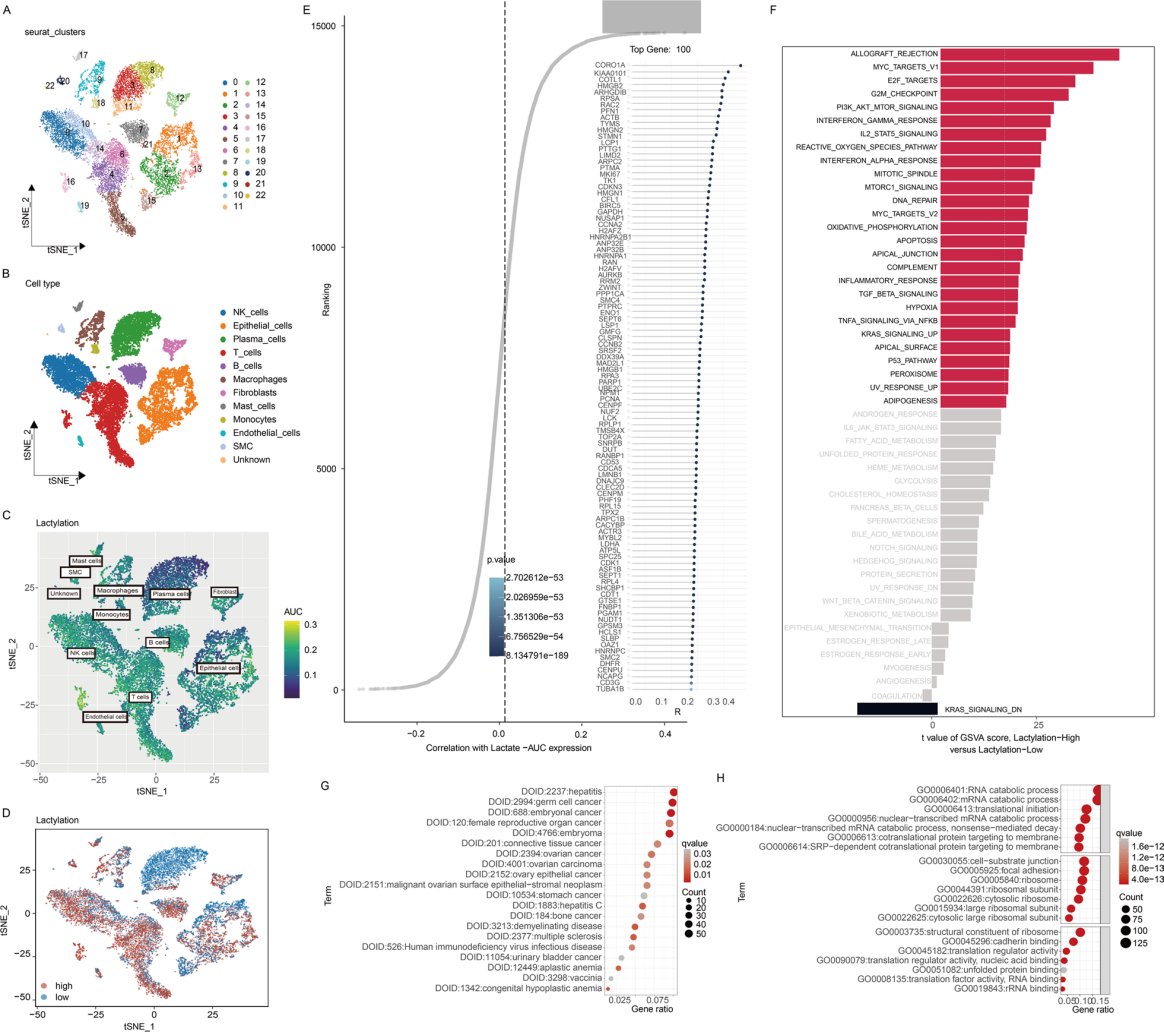
Identifying Lactylation-related genes (LRGs) in CRC from single-cell transcriptome. **A**, **B** The t-SNE plot illustrates the comprehensive annotation of CRC samples into 11 distinct cell types within TME. Each color on the plot corresponds to a specific cell type, as indicated. **C** The AUCell score, representing lactylation abundance in individual cells, is visually depicted in a gradient color scheme. **D** AUCell groups reflecting lactylation activity in each cell are projected, with red denoting the high lactylation group and blue indicating the low lactylation group. **E** Correlation analysis between lactylation-AUCell score and genes and the top 100 associated genes is presented according to R. p-values are displayed using a gradient of blue. **F** Barplot of GSVA sorted by t value highlights pathways that are upregulated in the high lactylation group. **G**, **H** Dotplot visualization demonstrates Gene Ontology (GO) and Disease Ontology (DO) biological process terms enriched in the high lactylation group. The color of the plots is determined by the q-value, with a gradient from low to high q-values

In order to identify genes mostly reflective of lactylation modification, we conducted deep analysis in single-cell sequencing data. After quality control, 46,286 high-quality cells were meticulously chosen for subsequent analysis. The expression profiles of each sample are visually depicted in Additional file 1: Fig. S2A. Principal component analysis (PCA) reduction plot indicated a lack of discernible differences in cell cycles, as illustrated in Additional file 1: Fig. S2C. The study encompassed 10 samples, and it was evident that the cell distribution within each sample remained remarkably consistent, suggesting the absence of significant batch effects among samples. Subsequently, dimensionality reduction techniques, specifically t-distributed stochastic neighbor embedding (t-SNE), successfully clustered all cells into 22 distinct groups, as shown in Fig. 3A. Bubble plots were employed to visualize the characteristic marker genes of various cell types as demonstrated in Additional file 1: Fig. S2F. The distribution of 11 specific cell clusters was visualized by t-SNE (Fig. 3B) and UMAP (Additional file 1: Fig. S2G, H). Based on the median AUC scores, all cells were assigned an AUC score for LRGs and categorized into high-lactylation-AUC and low-lactylation-AUC group (Fig. 3C). It was evident that cells with a higher number of lactylation-related genes (LRGs) tended to be predominantly red-colored immune cells, mainly NK, T cells, and B cells (Fig. 3D). Subsequently, we identified 879 differentially expressed genes (DEGs) between the high and low lactylation groups (Additional file 2: Table S5). Furthermore, a correlation analysis revealed the genes most strongly associated with lactylation activity (Fig. 3E) and we chose the top 100 highly lactylation-related genes, which we termed CORGs (Additional file 2: Table S6). In conclusion, this single-cell study identified 918 lactylation-related genes (LRGs) with the strongest links to lactylation activity by overlapping DEGs and CORGs. We further investigated LRGs-associated molecular signatures through Gene Set Variation Analysis (GSVA), Gene Ontology (GO) and Disease Ontology (DO) analysis at the single-cell level. The analysis indicated that lactylation played vital roles in pro-tumor pathways like “MYC,” “PI3K-AKT-mTOR,” “HYPOXIA” and was associated with immune-related pathways such as “INTERFERON_GAMMA_RESPONSE”, “IL2_STAT5_SIGNALING” and “TGF_BETA_SIGNALING” (Fig. 3F-H, Additional file 2: Tables S8–S10).

### Novel LRG signature unveils strong predictive power for CRC prognosis

**Fig. 4:**
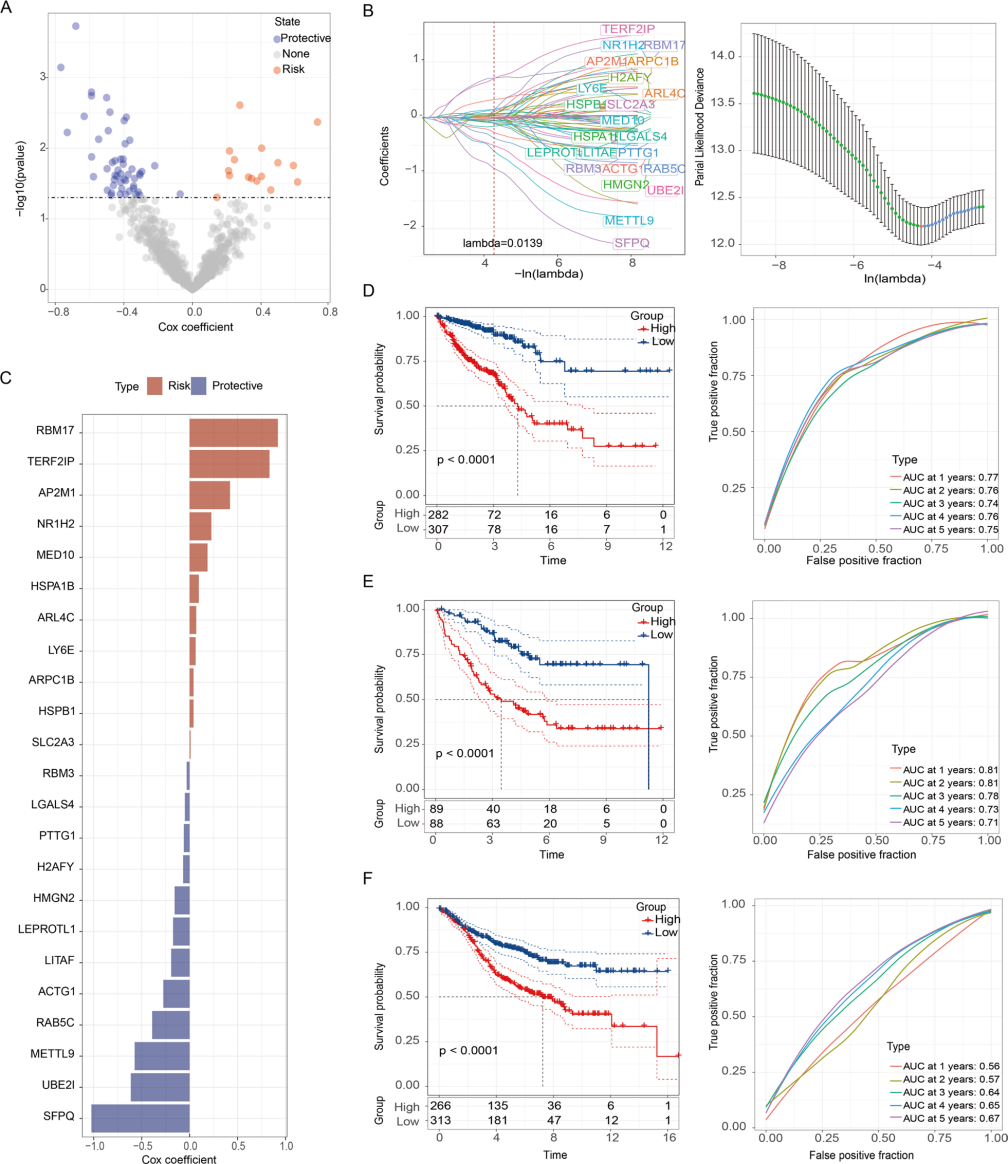
Novel LRG signature unveils strong predictive power for CRC prognosis. **A** Volcano plot illustrating prognostic-associated LRGs identified through univariate Cox proportional hazards analysis. **B** LASSO regression analysis included 23 prognostic LRGs to identify the most critical model genes. **C** Coefficients for model genes determined by multiple Cox proportional hazard ratios. Red indicates risk genes, and blue indicates protective genes. **D** Kaplan–Meier curves of Overall Survival (OS) based on the LRG risk signature in the TCGA dataset, accompanied by a time-dependent ROC curve demonstrating the survival accuracy of the model. **E**, **F** Kaplan–Meier curves of OS based on the LRG risk signature in the GEO datasets (GSE39582 and GSE17536), with a time-dependent ROC curve illustrating the survival accuracy of the model

Given the correlation between LRGs and pro-tumor pathways, we are intrigued by the possibility of their role in predicting CRC prognosis. Due to the limited sample size of scRNA-seq data, we opted to further analyze the TCGA cohort for the extraction of a LRG prognosis signature. Initially, a univariable Cox regression analysis identified 67 candidate genes associated with CRC, as depicted in the heatmap (Fig. 4A). To refine our gene selection, LASSO analysis (Fig. 4B) was employed, resulting in the identification of 23 genes with non-zero LASSO regression coefficients, including RBM17, TERF2IP, AP2M1, NR1H2, MED10, HSPA1B, ARL4C, LY6E, ARPC1B, HSPB1, SLC2A3, RBM3, LGALS4, PTTG1, H2AFY, HMGN2, LEPROTL1, LITAF, ACTG1, RAB5C, METTL9, UBE2I, SFPQ. These genes formed the basis of a predictive model termed the LRG score (LRGS) model (Fig. 4C). The LRGS model was constructed using the assigned coefficients for each gene, revealing a distinctive pattern where the initial 11 genes were associated with higher risk, while the subsequent 12 genes were linked to lower risk. This comprehensive model integrates the predictive power of these individual genes to offer a refined tool for evaluating CRC prognosis. The risk-score model was calculated according to the following equation:

$$\begin{aligned} {\text{risk score}} = & \left( {0.095837} \right) \times {\text{HSPA1B}} + \left( {0.040909} \right) \times {\text{HSPB1}} + \left( {0.04091} \right) \times {\text{ARPC1B}} \\ {\text{ }} & + \left( {0.010171} \right) \times {\text{SLC2A3}} + \left( {0.062486} \right) \times {\text{LY6E}} + \left( {0.069568} \right) \times {\text{ARL4C }} \\ & + \left( {0.420943} \right) \times {\text{AP2M1}} + \left( {0.832421} \right) \times {\text{TERF2IP}} + \left( {0.186926} \right) \times {\text{MED1}}0{\text{ }} \\ & + \left( {0.920787} \right) \times {\text{RBM17}} + \left( {0.226745} \right) \times {\text{NR1H2}} - \left( {0.04857} \right) \times {\text{LGALS4}} \\ \, - \left( {0.27447} \right) \times {\text{ACTG1}} - \left( {0.1725} \right) \times {\text{LEPROTL1}} - \left( {0.1566} \right) \times {\text{HMGN2}} \\ & - \left( {1.0237} \right) \times {\text{SFPQ}} - \left( {0.03022} \right) \times {\text{RBM3}} - \left( {0.61333} \right) \times {\text{UBE2I }} \\ & - \left( {0.0{\text{6724}}} \right) \times {\text{H2AFY}} - \left( {0.38982} \right) \times {\text{RAB5C}} - \left( {0.19226} \right) \times {\text{LITAF}} \\ & - \left( {0.57297} \right) \times {\text{METTL9}} - \left( {0.0604} \right) \times {\text{PTTG1}} \\ \end{aligned}$$

The prognosis value of the LRG signature was assessed by categorizing patients into high- and low-risk groups based on the median risk score. Kaplan–Meier survival analysis demonstrated a substantial variation between two groups in overall survival (OS) among TCGA-CRC patients and CRC patients from GSE39582 and GSE17536 where high-risk was equal to worse survival. Furthermore, ROC analysis was performed to evaluate the discriminative power of this signature. The 1-, 2-, 3-, 4-, and 5-year AUC values demonstrated strong predictive capabilities for the TCGA training set, GSE39582 test set, and GSE17536 test set (Fig. 4D–F). In all, this LRGS model stands out as a robust and promising tool for predicting CRC prognosis.

### Validation of the expression patterns of central genes in the LRGS

**Fig. 5:**
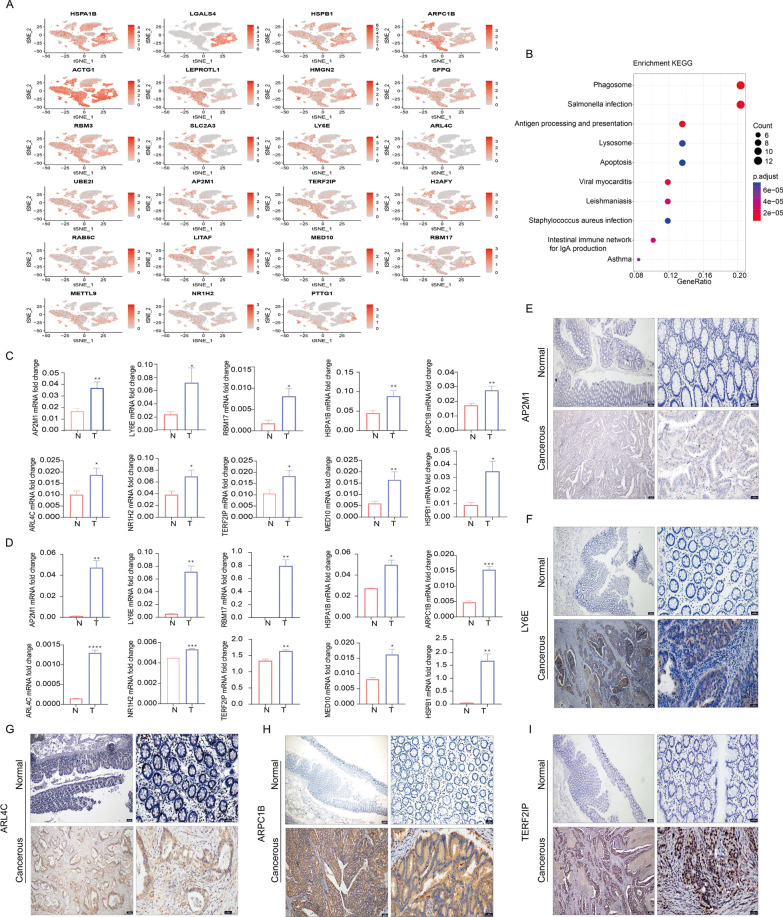
Validation of the expression patterns of central genes in the LRGS. **A** The t-SNE plot demonstrates the expression patterns of essential genes within the LRGS across distinct cell types based on single-cell RNAseq. **B** Functional enrichment analysis (GO analysis) highlighting the biological processes associated with the central genes of the LRGS in single-cell transcriptomic data. **C** Comparative qPCR analysis illustrating the expression disparities of LRGS risk genes, including AP2M1, LY6E, RBM17, HSPA1B, ARPC1B, ARL4C, NR1H2, TERF2IP, MED10, and HSPB1, between normal colon epithelial cell NCM460 (N) and various CRC cell lines including HCT8, HCT15, HCT116, RKO, DLD1, SW480, SW620 (T). **D** Comparative qPCR analysis showcasing the differential expression of LRGS risk genes in adjacent normal tissues (N) and cancerous tissues (T). N = 20 samples for each group and data are representative of three independent experiments. Error bars indicate mean ± SEM. **E**–**I** Representative IHC images of protein expression for AP2M1 (**E**), LY6E (**F**), ARL4C (**G**), ARPC1B (**H**), and TERF2IP (**I**) in normal and cancerous tissues. Scale bar: left panel, 200 μm; right panel, 50 μm. Statistical significance: *p < 0.05, **p < 0.01, ***p < 0.001, ****p < 0.0001

To further conform the distribution of these lactylation genes constituted LRGS in the tumor microenvironment (TME), we reanalyzed scRNA-seq data. The results showed that HSPB1, ACTG1, SFPQ, RBM3, LY6E, AP2M1, RAB5C, MED10, RBM17, METTL9, LGALS4 were predominantly expressed in epithelial cells and endothelial cells, while LEPROTL1, TERF2IP were prevailed in T and NK cells, ARL4C, ARPC1B, SLC2A3, H2AFY, LITAF was prominent in monocyte macrophages (Fig. 5A). Besides, NR1H2, PTTG1 were detected at low level in all cell clusters. KEGG enrichment analysis of 23 LRGs from single cell level also indicated that LRGs were associated with phagosome, antigen processing and presentation and so on (Fig. 5B, Additional file 2: Table S12). Subsequently, we turned to the bulk RNA transcriptome for further analysis. LEPROTL1, SLC2A3, LY6E, ARL4C, AP2M1, RAB5C, LITAF were positively related with immune score while LGALS4, SFPQ, RBM3, UBE2I, H2AFY, RBM17, PTTG1 were otherwise from estimate analysis, SLC2A3, LY6E were on the whole negatively associated with immune cell infiltration as we can see from the CIBERSORT analysis, while LITAF, ARL4C, SLC2A3 and LEPROTL1 were significantly correlated with almost all immune cells (Additional file 1: Fig. S3A–E). Subsequently, we conducted a comprehensive assessment of the expression levels of signature genes within various cell lines. Notably, the RNA expression levels of RBM17, TERF2IP, AP2M1, NR1H2, MED10, HSPA1B, ARL4C, LY6E, ARPC1B, HSPB1 were significantly elevated in CRC cell lines, such as HCT116, HCT15, HCT8, SW620, SW480, SW1116, RKO, and DLD1, in comparison to a normal cell line NCM460 (Fig. 5C). Additionally, to reinforce the significance of these gene expression alterations in CRC, we further scrutinized their levels in clinical tissues (Fig. 5D). Furthermore, our investigation extended to the protein expression levels of some select core risk genes, including AP2M1, LY6E, ARL4C, ARPC1B and TERF2IP by immunohistochemical analysis of CRC tissues alongside adjacent normal tissues which was in accordance with RNA level (Fig. 5E–I, Additional file 1: Fig. S3F). This additional layer of evidence underscores the relevance of these genes in CRC.

### Prediction of biological mechanisms associated with LRG signature

**Fig. 6:**
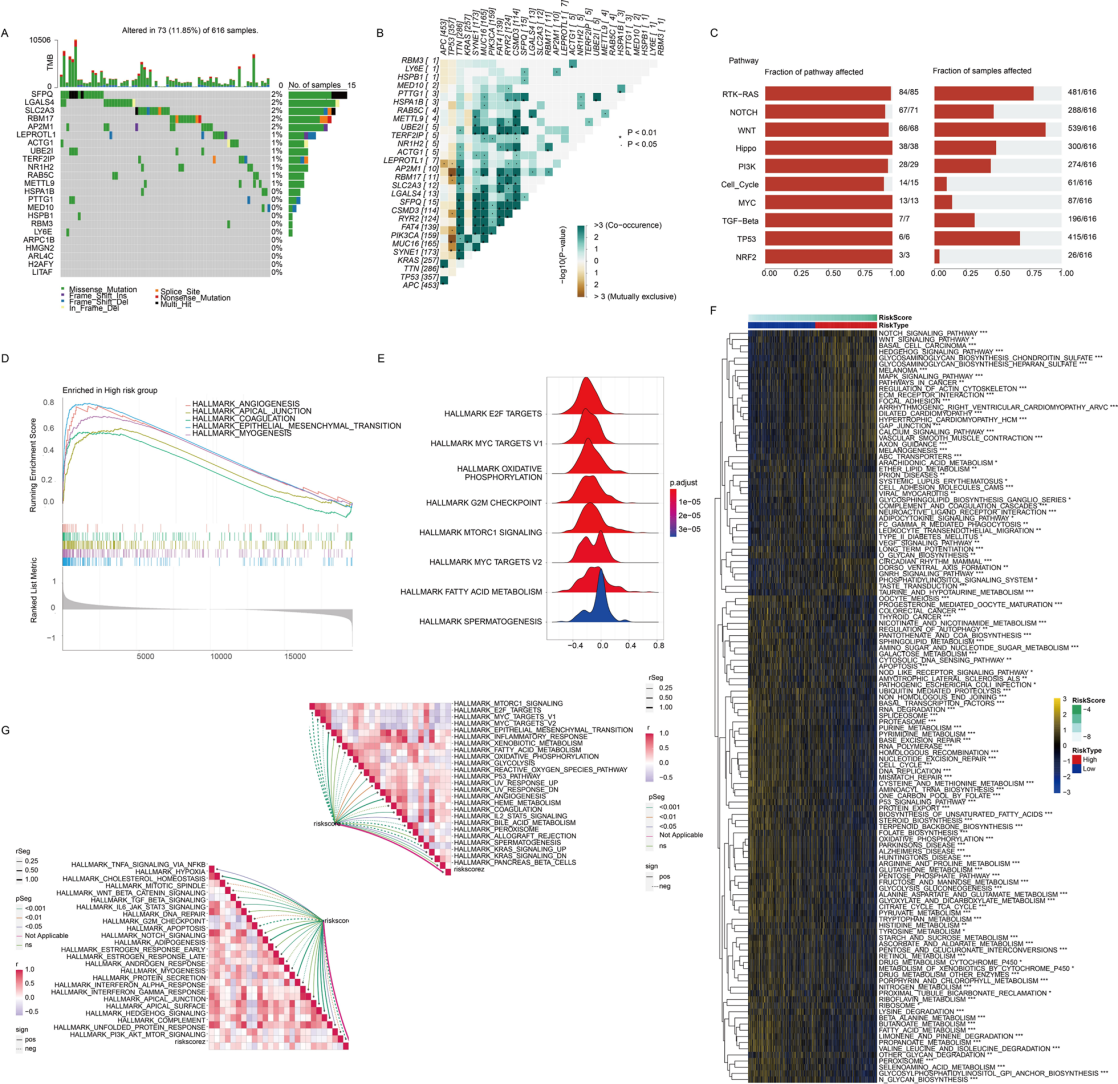
Prediction of biological mechanisms associated with LRGs risk. **A** Oncoprint visualization of the mutation atlas of these essential LRGs across CRC samples from TCGA cohort. **B** Co-mutation status between these essential LRGs and some commonly mutated genes. **C** The mutation frequencies of ten prevalent oncogenic pathways in high-risk group. **D** Identification of GO terms enriched in the high-risk group through GSEA analysis in TCGA. **E** Ridge plot showing the GO terms enriched in the low-risk group. **F** Assessment of variations in hallmark pathway activities between high- and low-risk groups based on GSVA scores. **G** Evaluation of the correlation between the LRG risk score and the activities of hallmark pathway. Statistical significance: *p < 0.05, **p < 0.01, ***p < 0.001, ****p < 0.0001

To explore the molecular implications of transcriptomic and genetic variances between high- and low-risk groups and gain insights into the biological processes linked to the poor survival in high-risk group, we delved into the genomic heterogeneity of the LRGS model within the TCGA cohort. Our examination of the mutation landscape of LRGs and the correlation analysis with frequently mutated genes like APC, TP53, TTN, and KRAS (Fig. 6A, B) revealed that LRGs did not carry a substantial mutation burden but were strongly associated with pro-tumor pathways, such as “RTK-RAS,” “WNT,” “Hippo,” “PI3K,” “MYC,” and immune-related pathways like “TGF-β” (Fig. 6C). Subsequently, functional enrichment analysis was conducted to uncover processes contributing to the poor prognosis of high-risk patients. In the GSEA analysis based on the GO gene set, the high-risk group showed enrichment in “ANGIOGENESIS,” “APICAL JUNCTION,” “COAGULATION,” and “EPITHELIAL MESENCHYMAL TRANSITION” (Fig. 6D). In contrast, the low-risk group exhibited enrichment in pathways like “E2F TARGETS,” “MYC TARGETS,” “OXIDATIVE PHOSPHORYLATION,” “G2M CHECKPOINT,” and “MTORC1 SIGNALING” (Fig. 6E). The GSVA analysis revealed differential expression in 122 pathways based on the risk score. Notably, the high-risk group was positively correlated with pathways such as “NOTCH SIGNALING PATHWAY,” “WNT SIGNALING PATHWAY,” “ECM RECEPTOR INTERACTION,” “FOCAL ADHESION,” and “ETHER LIPID METABOLISM,” all contributing to tumor growth (Fig. 6F**,** Additional file 2: Table S13). In addition, we conducted a correlation analysis between hallmark pathway activities and LRG risk score, revealing distinct characteristics between the high-risk and low-risk groups which further validated that high risk was associated with epithelial mesenchymal transition, angiogenesis, hypoxia, notch signaling and played vital role in immune signaling such as IL2-STAT5 signaling, TGFβ signaling, IL6 Jak stat signaling, IFNγ signaling and so on (Fig. 6G).

### The LRG score reshape the immune cell infiltration landscape

**Fig. 7:**
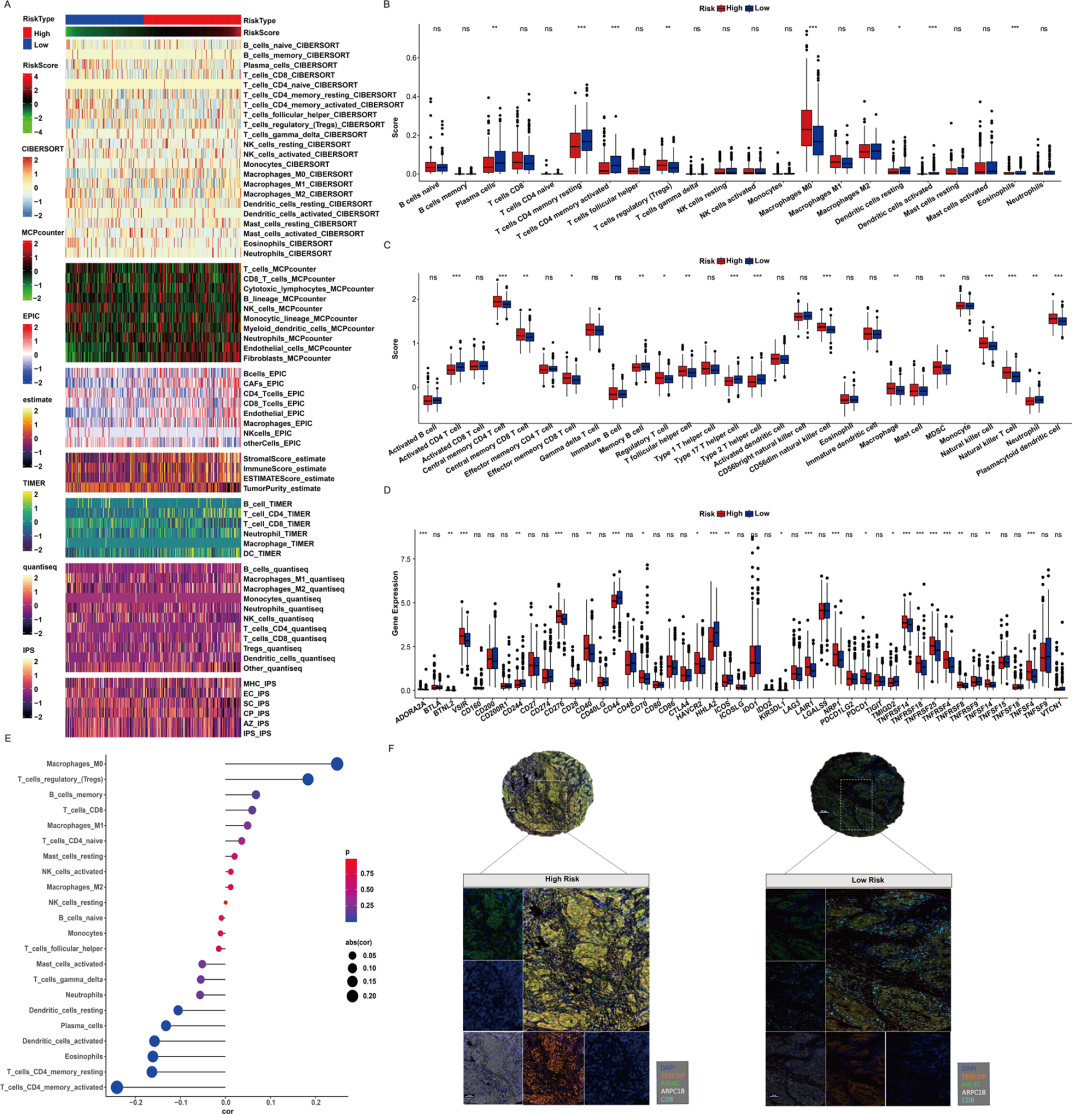
The LRG score reshape the immune cell infiltration landscape. **A** Seven algorithms assess differences in immune infiltration status between different risk groups. **B**, **C** (B-CIBERSORT; C-ssGSEA) Boxplot illustrating the relative abundance of infiltrating immune cell types in patients belonging to high and low-risk groups. **D** Boxplot of relative expression levels at 27 immune checkpoints profiles between the high and low risk patients. **E** Correlation analysis between TME-infltrated cells and LRGR. **F** Representative images of mIHC showed the relationship between TERF2IP, ARL4C, ARPC1B and CD8. Statistical significance: *p < 0.05, **p < 0.01, ***p < 0.001, ****p < 0.0001

Recent research has underscored the pivotal role of the inflammatory microenvironment in CRC development, particularly the activation status and interactions of immune and stromal cells with tumor cells [33, 34]. It’s reported that the lactylation level is closely related to immune signaling and contributes to the remodeling of the tumor environment [35]. Here, our comprehensive analysis, employing seven distinct algorithms, consistently demonstrates heightened T cell infiltration in low-risk tumors (Fig. 7A). Further, an evaluation of 22 immune cell landscapes through CIBERSORTx reveals significant differences in T cell subtypes, with CD4 memory cells showing lower infiltration and Treg and macrophages exhibiting higher infiltration in the high LRG score group (Fig. 7B). Additionally, dendritic cells, crucial antigen-presenting cells, are prevalent in the low-risk group. Single-sample Gene Set Enrichment Analysis (ssGSEA) reaffirms these findings, emphasizing the robust anti-tumor immune response in the low-risk group (Fig. 7C). Exploring the relationship between risk score and immunotherapy biomarkers, we observe elevated expression of Immune Checkpoint Genes (ICGs) like PD-1 and PDCD1 in the high-risk group, suggesting potential therapeutic targets (Fig. 7D). Validation through multiple Immunohistochemistry (mIHC) confirms the prominence of ARL4C, ARPC1B, and TERF2IP in tumor tissue, and their expression were negatively associated with CD8 T cell presence (Fig. 7E, F). This comprehensive analysis highlights the intricate interplay between lactylation level and immune cell infiltration, reinforces the relationship between cancer and TME, thus providing potential for immunotherapy insights in CRC.

### Predictive value of LRG signature for immunotherapy

**Fig. 8:**
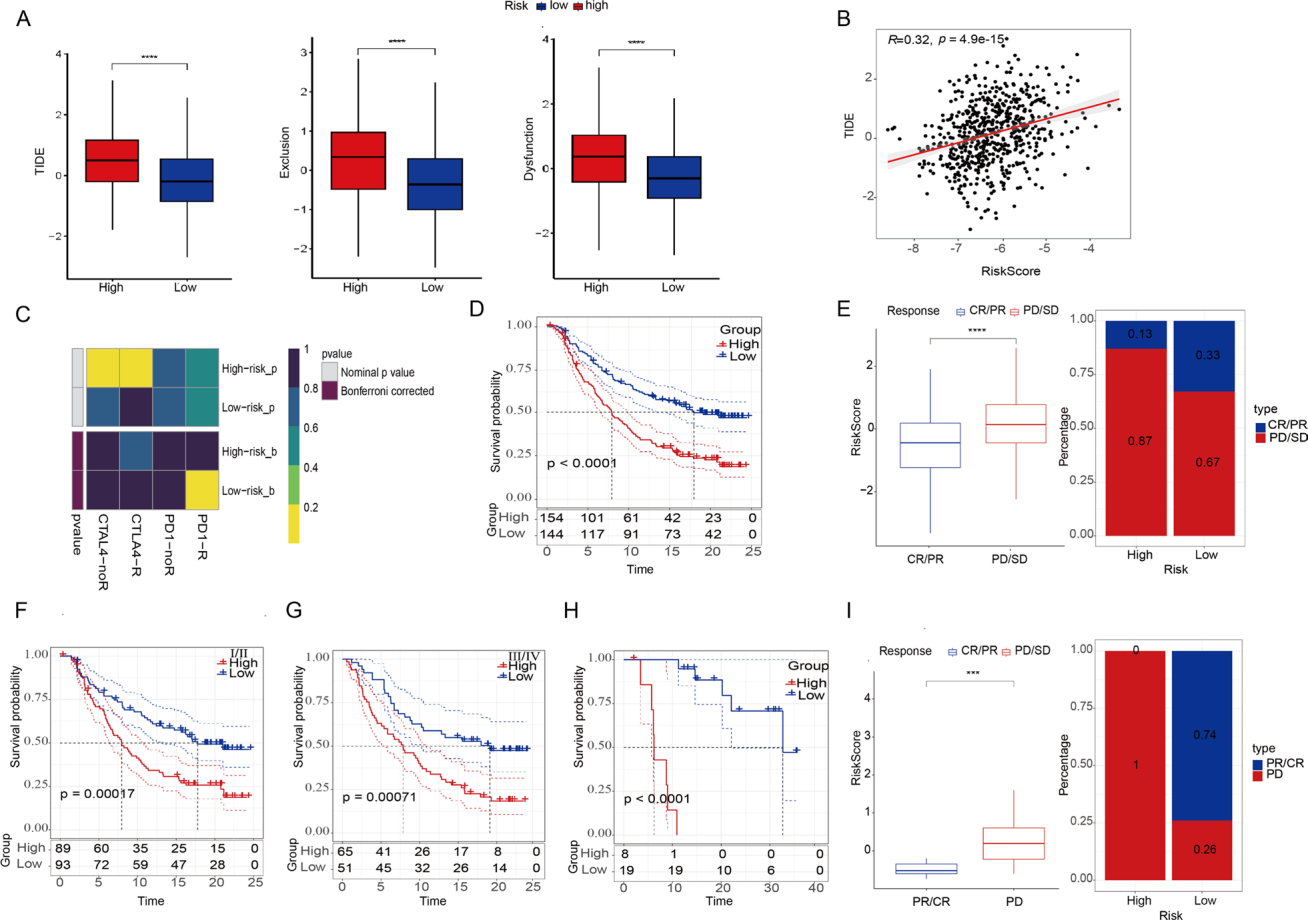
Predictive value of LRG signature for immunotherapy. **A** Boxplot of TIDE score between the high- and low-risk group. A higher score indicates enhanced probability of immune invasion. **B** Scatter plot of ESTIMATE indicates a positive relationship between risk score and TIDE score. **C** Heatmap of Submap analysis of the two groups (Bonferroni-corrected p < 0.05). For Submap analysis, a smaller p-value implied a more similarity of paired expression profile. **D**–**G** survival curve (**D**) and immunotherapy response (**E**) between high risk and low risk group in IMvigor210 cohort and survival curve of patients at stage I/II (**F**) and III/IV (**G**). **H**, **I**. survival curve (**H**) and immunotherapy response (**I**) between high risk and low risk group in GSE78220 cohort. Statistical significance: *p < 0.05, **p < 0.01, ***p < 0.001, ****p < 0.0001

Expanding on the pivotal role of immune infiltration in disease progression and response to immunotherapies, alongside the influence of lactylation in reshaping TME, we investigated the predictive capacity of our prognostic model for CRC patients undergoing ICI treatment. Utilizing the TIDE score, we observed a significant increase in the Exclusion score, Dysfunction score, and overall TIDE score in the high-risk group (Fig. 8A, B). This suggests a heightened potential for immune escape among high-risk patients, possibly leading to reduced effectiveness of ICI therapy. Subclass Mapping (Submap) results further indicated that the low-risk group might be more sensitive to PD-1 therapy (Bonferroni-corrected p < 0.05) (Fig. 8C**)**. Furthermore, we validate the efficiency across multiple immunotherapy datasets, the IMvigor dataset demonstrated better survival outcomes (Fig. 8D) and improved responses to anti-PD-L1 immunotherapy (Fig. 8E) in the low-risk group. This trend persisted across different stages, with low-risk patients exhibiting better prognosis in both stage I or II (Fig. 8F) and stage III or IV (Fig. 8G). Consistent results were obtained in the GSE78220 dataset, further confirming the low-risk group's favorable response to immunotherapy (Fig. 8H, I). This comprehensive analysis underscores the potential of our prognostic model in predicting immunotherapy outcomes for CRC patients.

### A nomogram integrating LRG Score and clinical parameters for precision prediction

**Fig. 9:**
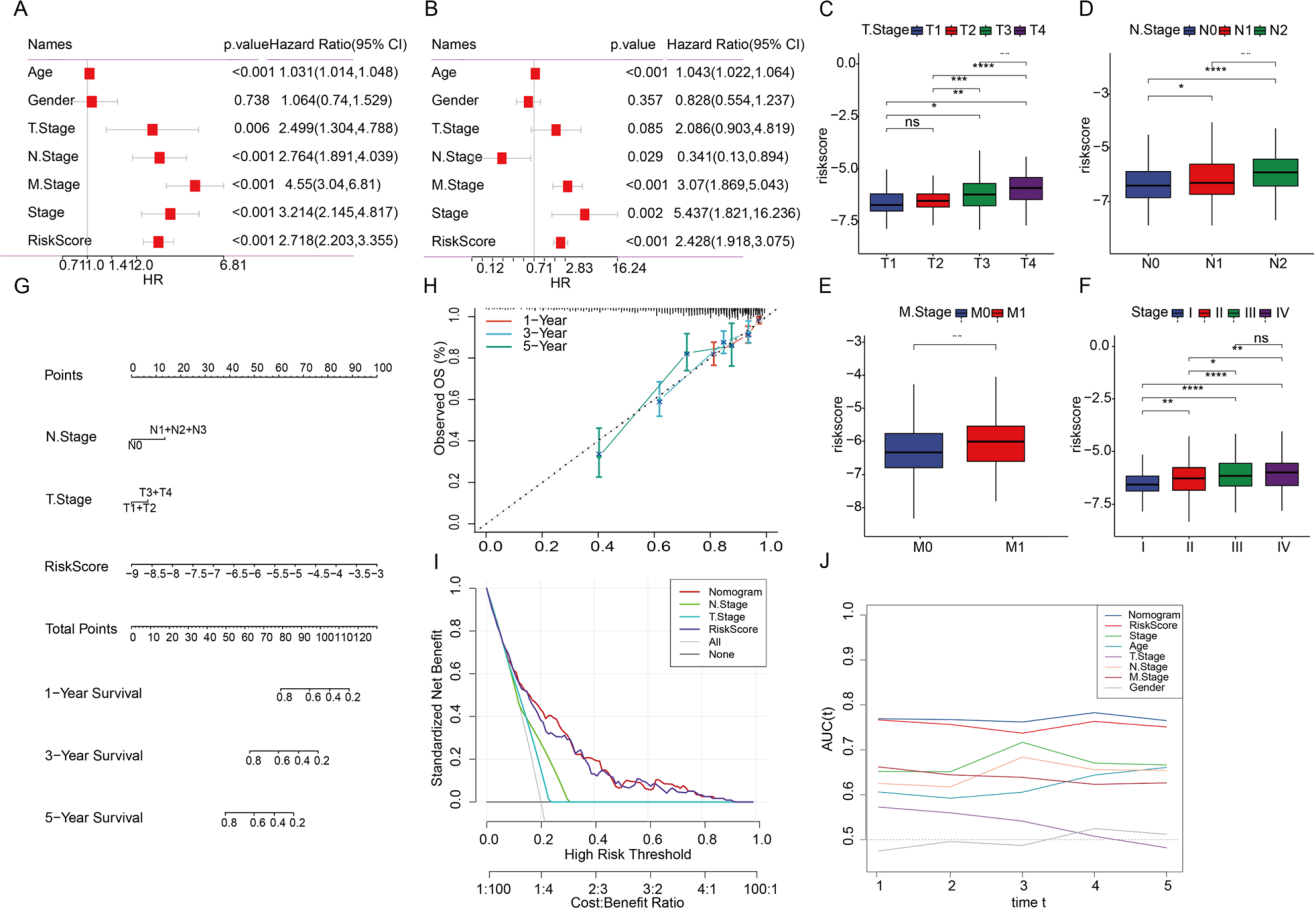
A nomogram integrating LRG Score and clinical parameters for precision prediction. **A** Univariate independent prognostic analysis in the TCGA cohort. **B** Multivariate independent prognostic analysis in the TCGA cohort. **C**–**F** Barplot shows the relationship between different clinical factors and LRG riskscore. **G** Nomogram was constructed by combining clinical features (T stage, N stage) with risk score. **H** The calibration plots test consistency between the actual OS rates and the predicted survival rates, with the 45°line representing the best prediction. **I** Decision curve analysis (DCA) showed that nomograms had a significantly higher clinical benefit for patients than other clinical features. **J** AUC curves were used to evaluate the predictive performance of different clinical characteristics, nomogram scores and risk scores. Statistical significance: *p < 0.05, **p < 0.01, ***p < 0.001, ****p < 0.0001

In the pursuit of other clinical applicability for the LRG score, we conducted independent and multiple prognostic analyses to identify potential factors influencing the prognosis of CRC and confirmed that LRGR was an independent risk factor of CRC (Fig. 9A, B). Visualizing the correlations between model genes and various clinical characteristics, we observed a higher prevalence of unfavorable T stage, N stage, M stage, and clinical stage values in the high-risk group, while no significant association was found with age and gender (Fig. 9C–F, Additional file 1: Fig. S4). Using the TCGA-CRC dataset, we meticulously developed a predictive nomogram which combines the risk score with crucial clinicopathological parameters such as T stage and N stage to predict 1-, 3-, and 5-year OS rates (Fig. 9G). Due to the scarcity of samples with M stage information, we excluded M stage from the nomogram. Calibration curves confirmed the accuracy of the nomogram aligned with actual outcomes in 1-, 2-, and 3-year predictions (Fig. 9H). Decision Curve Analysis (DCA) demonstrated the nomogram's superiority over using LRGS alone in terms of clinical benefits for patients (Fig. 9I). Time-dependent AUC further substantiated the nomogram's superior predictive performance compared to the risk score and other conventional clinical measures (Fig. 9J).

### Seeking potential therapeutic agents for the high LRGS group

**Fig. 10:**
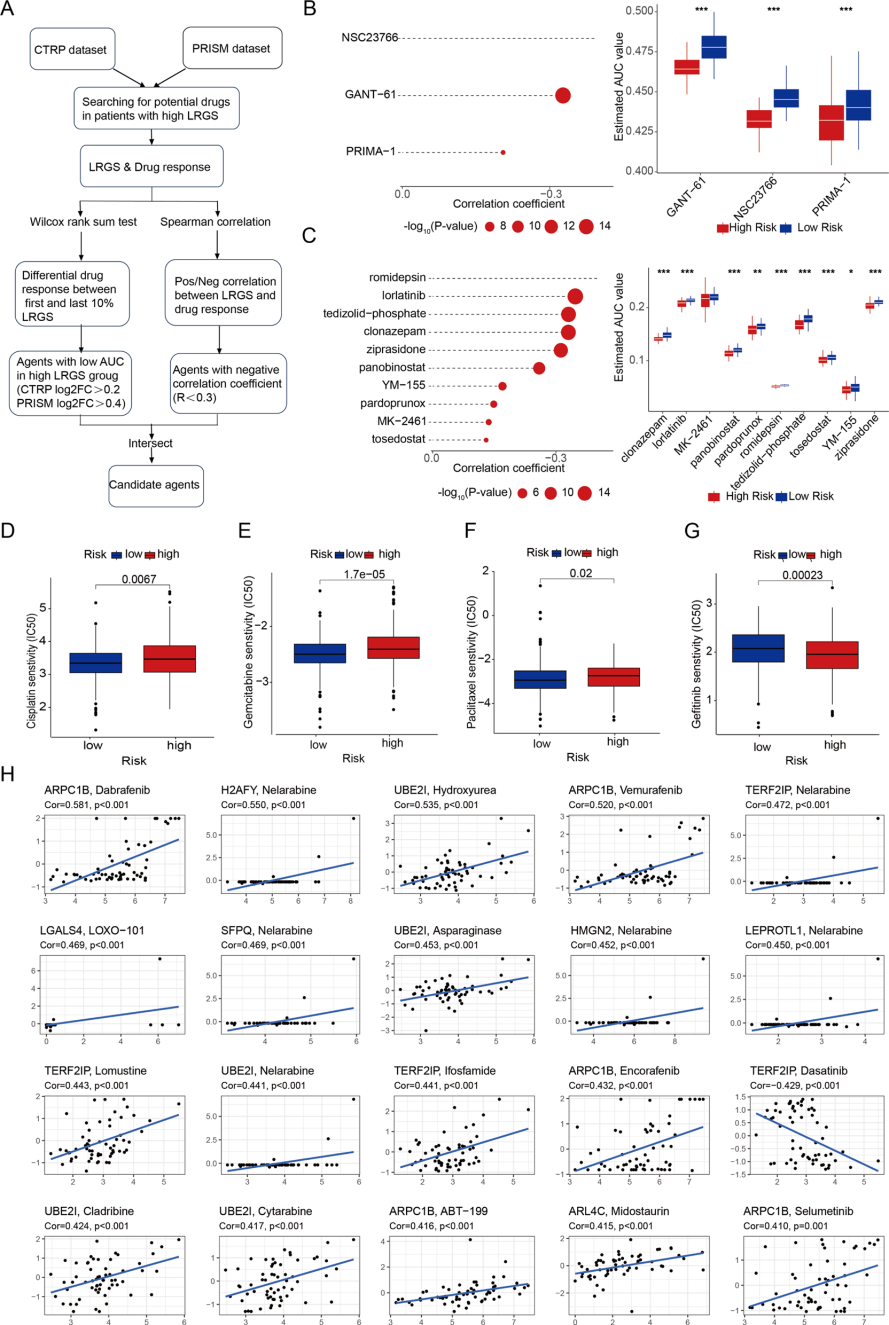
Seeking potential therapeutic agents for the high LRGS group. **A** Schematic outlining the strategy to develop potential therapeutic agents with higher drug sensitivity in the high risk group. **B** The results of Spearman’s correlation analysis and differential drug response analysis of three CTRP-derived compounds. **C** The results of Spearman’s correlation analysis and differential drug response analysis of ten PRISM-derived compounds. Lower AUC values imply greater sensitivity. D-G. The differences of sensitivity of patients to cisplatin (**D**), Gemcitabine (**E**), paclitaxel (**F**) and Gefitnib (**G**). Lower IC50 is equal to better sensitivity. **H** Scatter plot illustrating spearman’s correlations and significance between the 23 model genes and chemotherapeutic drugs using data from the CCLE. Statistical significance: *p < 0.05, **p < 0.01, ***p < 0.001, ****p < 0.0001

To identify potential therapeutic options for CRC patients with high LRGS, our study employed a comprehensive analysis of chemical and targeted drugs. Utilizing sensitivity data from the Cancer Therapeutics Response Portal (CTRP) and Profiling Relative Inhibition Simultaneously in Mixtures (PRISM) datasets, we identified promising agents specifically for the high LRGS group. From the CTRP dataset, GANT-61, NSC23766, and PRIMA-1 emerged as potential candidates (Fig. 10B), while the PRISM dataset highlighted romidepsin, lorlatinib, tedizolid-phosphate, and others (Fig. 10C). The AUC values for these agents were significantly lower in the high LRGS group, indicating better sensitivity, with detailed drug functions provided in the Additional file 2: Table S14. Furthermore, we evaluated the half-maximal inhibitory concentration (IC50) for four chemotherapeutic drugs in two risk groups. In the low-risk group, IC50 values for cisplatin, gemcitabine, and paclitaxel were significantly lower than those in the high-risk group, suggesting potential benefits for low-risk patients. Conversely, gefitinib showed lower IC50 in the high-risk group, making it a more suitable option for this group (Fig. 10D-G). Nine LRGS-associated genes, including ARPC1B, TERF2IP, ARL4C, UBE2I, LGALS4, SFPQ, HMGN2, LEPROTL1, and H2AFY, were linked to the sensitivity of specific chemotherapeutic drugs (p < 0.05) (Fig. 10H). These genes encompass three risk genes in LRGS, namely ARPC1B, TERF2IP, and ARL4C and six protective genes, including UBE2I, LGALS4, SFPQ, HMGN2, LEPROTL1, and H2AFY. ARPC1B and UBE2I exhibited broad associations with various drugs. For instance, increased expression of ARPC1B was linked to enhanced sensitivity to Dabrafenib, Vemurafenib, Encorafenib, ABT-199, and Selumetinib in CRC patients. Similarly, elevated UBE2I expression was related to increased sensitivity to Nelarabine, Hydroxyurea, Asparaginase, Cladribine, and Cytarabine. Among the risk genes, TERF2IP ranked second in terms of associations with Nelarabine, Lomustine, Ifosfamide, and Dasatinib. These findings suggest intricate interactions between LRGs and drug sensitivities in cancer therapy (Additional file 2: Table S15) and thereby provide more directions for clinical usage in the near future.

## Discussion

Lactate, once considered a metabolic waste product, has emerged as a key player in tumor metabolic reprogramming, intricately shaping TME and directly influencing immune cells. A growing body of research is now dedicated to unraveling the intricate interplay between lactate and diverse immune cells in the tumor microenvironment, with the aim of improving the efficacy of existing anti-tumor immune therapies [36]. Prospective studies on lactylation and in-depth immune metabolism hold the promise of unveiling new drugs that can selectively regulate immune cell activity with minimized side effects. Despite significant research on the importance of lactylation in cancer prognosis and treatment across various tumor types such as lung cancer and hepatocellular carcinoma [22] and gastric cancer [37], research on lactylation modifications in CRC is limited, and no lactylation profile or global lysine lactylation identification has been established.

In our study, we innovatively demonstrated a markedly elevated level of lactylation in paired tumor tissues compared to normal tissues, as well as in CRC cell lines compared to normal epithelial cell NCM460. This discovery prompted a focused investigation into lactylation modification, subsequently confirming its association with CRC progression and establishing it as an independent prognostic factor in CRC. Therefore, a comprehensive understanding of how lactylation functions in cancer initiation and progression is imperative for its potential application in diagnosis and prognosis. Utilizing published single-cell RNA sequencing (scRNA-seq) data, we identified 918 genes pivotal in regulating lactylation levels, with T cells and NK cells exhibiting a high association with lactylation activity. Combining this with bulk RNA-seq data containing 586 CRC patients with prognosis information, we developed a prognosis model comprising 23 core genes. This model was rigorously validated using data from 756 patients through survival plots and ROC curves. Moreover, we observed that the risk score significantly correlated with the TNM staging system, prompting the construction of a predictive nomogram encompassing the risk score and clinicopathological parameters (T stage and N stage). This nomogram outperformed other clinical parameters, showcasing its potential clinical utility. Despite the low levels of gene alterations, the identified pattern associated with pro-tumor and immunoregulatory pathways suggests the influence of metabolites. Our study proposes targeting pathways like PI3K-AKT-mTOR, HYPOXIA, and MYC, providing intriguing opportunities for therapeutic intervention. Building upon previous findings that lactate in the TME exerts a substantial inhibitory influence on immune cells, particularly anti-tumor T cells, we observed a similar phenomenon in the high-risk group, negatively impacting CRC prognosis. This was attributed, in part, to the infiltration of immunosuppressive T cells like Tregs and a reduction in activated CD8 T cells. Gene interventions modifying glucose metabolism to enhance the TME could serve as valuable adjuncts, especially in conjunction with prevalent immunotherapies like immune checkpoint inhibitors (ICIs). A deeper understanding of gene localization and control mechanisms influencing immune cell function within the tumor microenvironment will guide the selection of optimal immunotherapies. Furthermore, our selection of vital genes for expression analysis between tumor and normal tissues, along with their interaction with the tumor environment, provides valuable targets for future lactylation research in CRC. We extended our analysis to assess the response to immunotherapy between the two risk groups, aligning with our earlier findings that the high-risk group exhibits a low response to immunotherapy. Validation of this conclusion in an immunotherapy cohort suggests potential applications for patient selection and transforming the cold tumor microenvironment into a responsive one. Additionally, we conducted an analysis of various chemical and targeted drugs, presenting promising results despite the absence of relevant in vivo or in vitro experimental validation. This opens new avenues for future investigations in CRC treatment.

### Novelty in lactylation research

This study delves into the emerging realm of lactylation within the landscape of CRC. By integrating diverse omics data types, including transcriptomic, genomic, and immunotherapy response data, our multi-dimensional approach enriches the analysis, providing a comprehensive perspective on lactylation in the context of colorectal cancer. This integrated methodology offers a more nuanced understanding of the molecular landscape, shedding light on the intricate world of post-translational modifications in cancer biology. A distinctive contribution of this research lies in the identification of lactylation-related genes and their intricate association with cancer. This novel insight significantly advances our comprehension of the molecular mechanisms underlying lactylation in colorectal cancer. The catalog of lactylation-related genes emerges as a valuable resource, empowering researchers and clinicians alike to delve into the molecular intricacies and potential therapeutic targets associated with lactylation. Furthermore, the prognostic value of these genes in predicting the survival outcomes of CRC patients introduces clinical implications, facilitating risk stratification and the formulation of personalized treatment strategies. The exploration of lactylation-related genes in the context of immunotherapy response is a notable aspect of this study. The insights gained could potentially influence patient selection in immunotherapy trials, guiding the development of targeted therapeutic strategies. This facet of the research carries promising implications for advancing precision medicine in colorectal cancer treatment. Moreover, the identification of drugs related to lactylation opens avenues for further experimental studies and clinical trials. This information serves as a compass, guiding researchers in evaluating the efficacy of these agents within the intricate landscape of colorectal cancer treatment. In essence, this study not only expands our knowledge of lactylation in CRC but also lays the groundwork for future advancements in both basic research and clinical applications.

### Limitations

While our lactylation-related signature underwent validation through multiple methods, certain limitations persist. Given the nascent state of research in protein lactylation, the precise impact on protein function within tumor cells remains elusive. The absence of dedicated lactylation antibodies and suitable antibodies for all model genes specialized fot IHC and mIHC poses a practical challenge for experimental validation of all signature genes. That’s the reason why we didn’t perform IHC and mIHC on all model genes. However, we anticipate that these challenges will diminish as our comprehension of lactylation advances. Future investigations should prioritize lactylation proteomic studies to broaden the spectrum of lactylation-related genes. Integrating protein expression data with corresponding lactylation modification data will enhance our insights. Moreover, the development of specific lactylation modification protein antibodies is recommended to explore functional effects on proteins and elucidate underlying mechanisms. Regrettably, we lacked an immunotherapy cohort or other public CRC datasets with immunotherapy prognosis to validate our conclusions, a gap that can be addressed with the growing prevalence of immunotherapy in CRC.

## Conclusion

In summary, our study unveils, for the first time, the significant impact of global lactylation on the clinical prognosis of CRC and identifies a distinct set of genes highly indicative of lactylation activity at the single-cell level. This groundbreaking discovery not only introduces a novel prognostic indicator for identifying CRC patients with poor outcomes but also present potential therapeutic targets and provide insights into the molecular mechanism in CRC patient. Employing this multi-dimensional approach, our research significantly broadens the spectrum of options available, offering unprecedented insights to maximize the efficacy of CRC therapy.

### Supplementary Information

**Additional file 1: Figure S1.** The presence of lactylation in CRC cell lines. **Figure S2.** Quality control visualization of 10 samples in single cell transcriptome. **Figure S3.** Specific locations and functions of 23 core genes. **Figure S4.** Prognostic value of the LRGS risk.

**Additional file 2: Table S1.** Lactylation related genes from previous research. **Table S2.** Top 10 marker genes in 22 clusters. **Table S3.** Marker gene reference for all cells. **Table S4.** Marker gene reference for immune cells. **Table S5.** DEGs identified from high lactylation and low lactylation group. **Table S6.** Top 100 Lactylation-Associated Genes (CORGs). **Table S7.** Lactylation-related-genes indentified from our study. **Table S8.** GSVA analysis between high lactylation and low lactylation group. **Table S9.** GO pathways enriched in high lactylayion cells. **Table S10.** DO pathways enriched in high lactylayion cells. **Table S11.** Construction of the LRGS model. **Table S12.** GO pathways enriched in 23 model genes. **Table S13.** GSVA analysis between high risk and low risk group. **Table S14.** Specific function of drugs screened from CTRP and PRISM. **Table S15.** Association between core genes and specific drugs. **Table S16.** Primers used in this study.

## Data Availability

The datasets used and/or analyzed during the current study are available from the corresponding author on reasonable request. The data that support the results of current study is available on TCGA (https://portal.gdc.cancer.gov/) and GEO websites (http://www.ncbi.nlm.nih.gov/geo).

## Abbreviations

CRC: Colorectal cancer
ICI: Immune checkpoint inhibitor
dMMR: Mismatch-repair-deficient
MSI-H: High levels of microsatellite instability
MSS: Microsatellite stable
pMMR: Mismatch-repair-proficient
PTM: Post-translational modifications
scRNA-seq: Single-cell RNA sequencing
TCGA: The Cancer Genome Atlas
GEO: Gene Expression Omnibus
LRGs: Lactylation related genes
HVG: Highly variable genes
PCA: Principal component analysis
DEGs: Differentially Expressed Genes
GSVA: Gene Set Variation Analysis
GO: Gene Ontology analysis
DO: Disease Ontology analysis
KEGG: Kyoto Encyclopedia of Genes and Genomes
GSEA: Gene set enrichment analysis
ssGSEA: Single sample gene set enrichment analysis
LASSO: Least absolute shrinkage and selection operator
LRGS: Lactylation related genes score
ROC: Receiver operating characteristic
AUC: Area under the curve
TMB: Tumor mutation burden
TIDE: Tumor immune dysfunction and exclusion
DCA: Decision curve analysis
CTSP: Cancer Therapeutics Response Portal
PRISM: Profiling Relative Inhibition Simultaneously in Mixtures
GDSC: Genomics of drug sensitivity in cancer database
CCLE: Cancer cell line encyclopedia
ATCC: American Type Culture Collection
TMA: Tumor tissue microarray
qRT-PCR: Quantitative real-time PCR
IHC: Immunohistochemistry
mIHC: Multiple immunohistochemistry
DAB: 3,3-diaminobenzidine
SD: Standard deviation
TME: Tumor microenvironment
