# Supplementary material for: A multi-dimensional approach to unravel the intricacies of lactylation related signature for prognostic and therapeutic insight in colorectal cancer
Source: J Transl Med. 2024 Feb 28;22:211. doi: 10.1186/s12967-024-04955-9 (PMC10900655; doi:10.1186/s12967-024-04955-9)
Supplement: Supplementary file 1 — Additional file 1: Figure S1. The presence of lactylation in CRC cell lines. Figure S2. Quality control visualization of 10 samples in single cell transcriptome. Figure S3. Specific locations and functions of 23 core genes. Figure S4. Prognostic value of the LRGS risk. [file 12967_2024_4955_MOESM1_ESM.docx]

**Additional file Legends for**

**A multi-dimensional approach to unravel the intricacies of lactylation related signature for prognostic and therapeutic insight in colorectal cancer**

Huixia Huang^1,2,3,4,#^, Keji Chen^1,2,3,4,#^, Yifei Zhu^1,2,3,4,#^, Zijuan Hu^1,2,3,4^, Yaxian Wang^5,6^, Jiayu Chen^5,6^, Yuxue Li^5,6^, Dawei Li^5,6,*^, Ping Wei^1,2,3,4,*^

^1^Department of Oncology, Shanghai Medical College of Fudan University, Shanghai, China.

^2^Department of Pathology, Fudan University Shanghai Cancer Center, Shanghai, China.

^3^Cancer Institute, Fudan University Shanghai Cancer Center, Shanghai, China.

^4^Institute of Pathology, Fudan University, Shanghai, China.

^5^Department of Colorectal Surgery, Fudan University Shanghai Cancer Center, Shanghai, 200032, China.

^6^Department of Oncology, Shanghai Medical College Fudan University, Shanghai, China.

^*^Correspondence to: weiping@fudan.edu.cn (Ping Wei) or li_dawei@fudan.edu.cn (Dawei Li).

^#^These authors contributed equally to this work.

**This file includes supplementary Figure S1-4 and Captions for Supplementary Table 1-16**

**Additional file Figures and Legends**


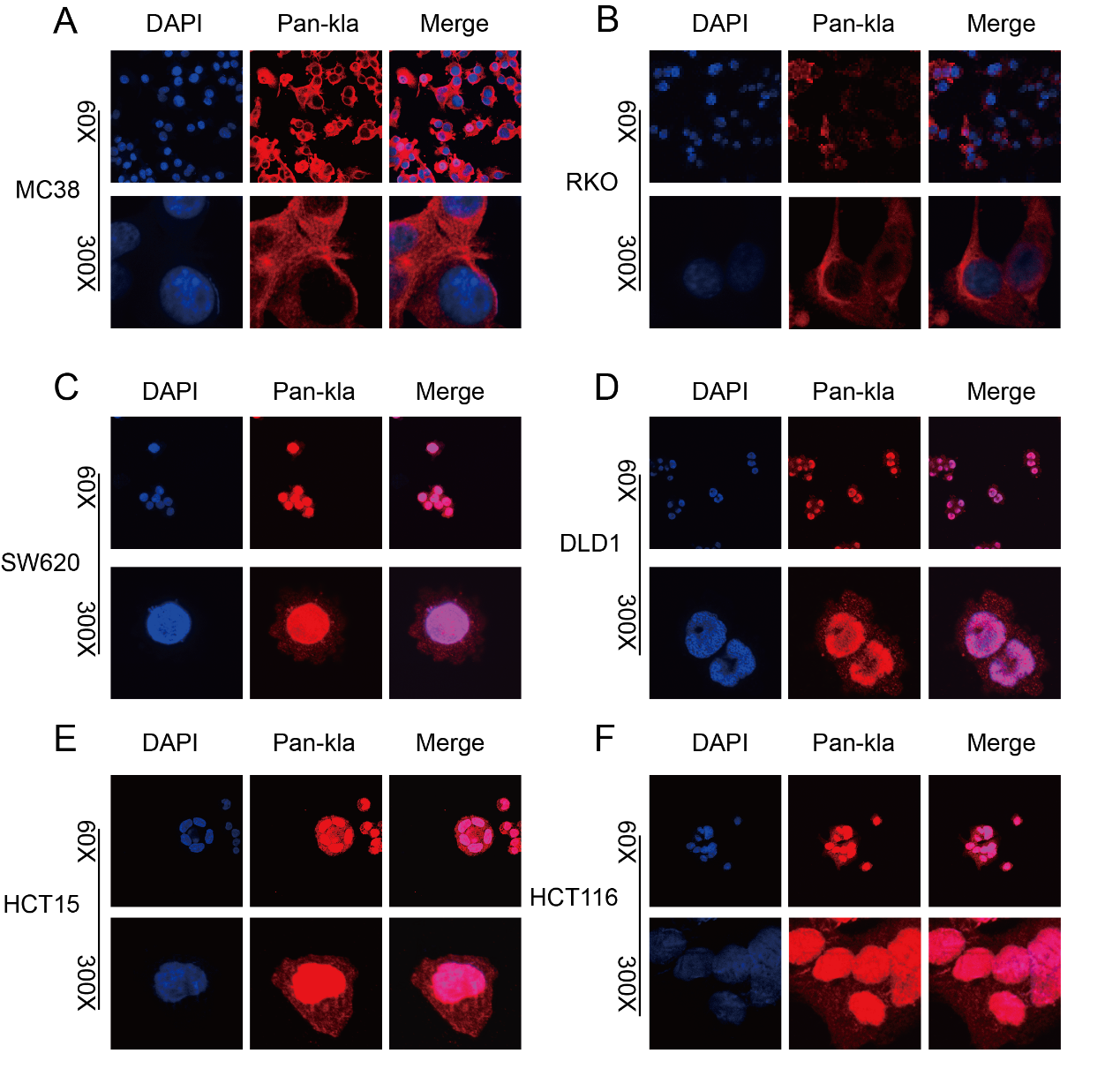


**Figure S1. The presence of lactylation in CRC cell lines.**

A-F. Representative IF images of pan-Kla in murine CRC cell line MC38 and human counterparts RKO, SW620, DLD1, HCT15 and HCT116


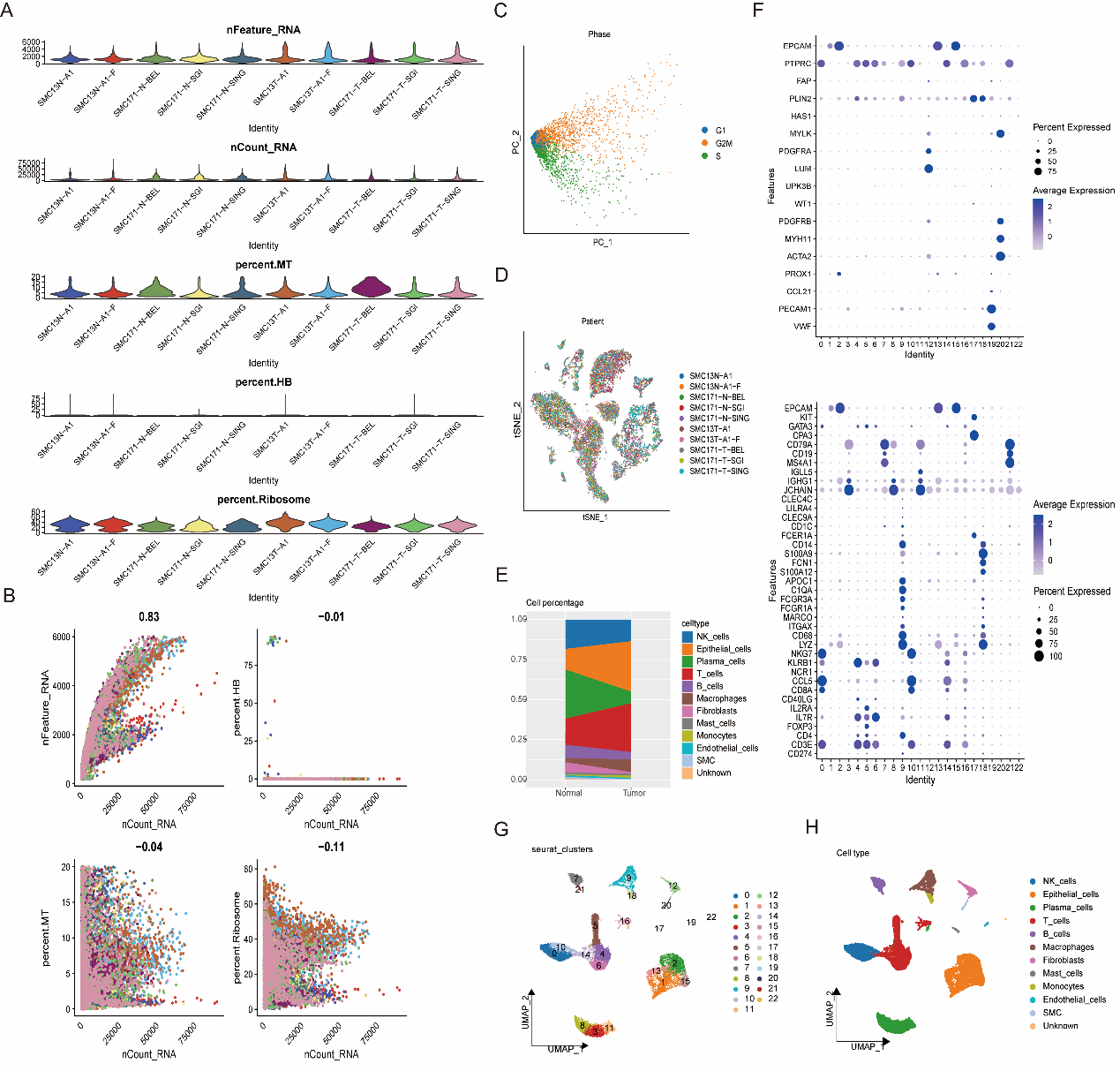


**Figure S2. Quality control visualization of 10 samples in single cell transcriptome.**

A-B. Quality Control Assessment of Single-Cell Transcriptome

C. Principal Component Analysis (PCA) Plot

D. tSNE Plot of the Samples

E. Cell Percentage Distribution across 12 Clusters in Tumor and Normal Samples

F. Bubble Plot Illustrating Marker Genes for 22 Clusters

G-H. Uniform Manifold Approximation and Projection (UMAP) Plot of 11 Combined Cell Clusters


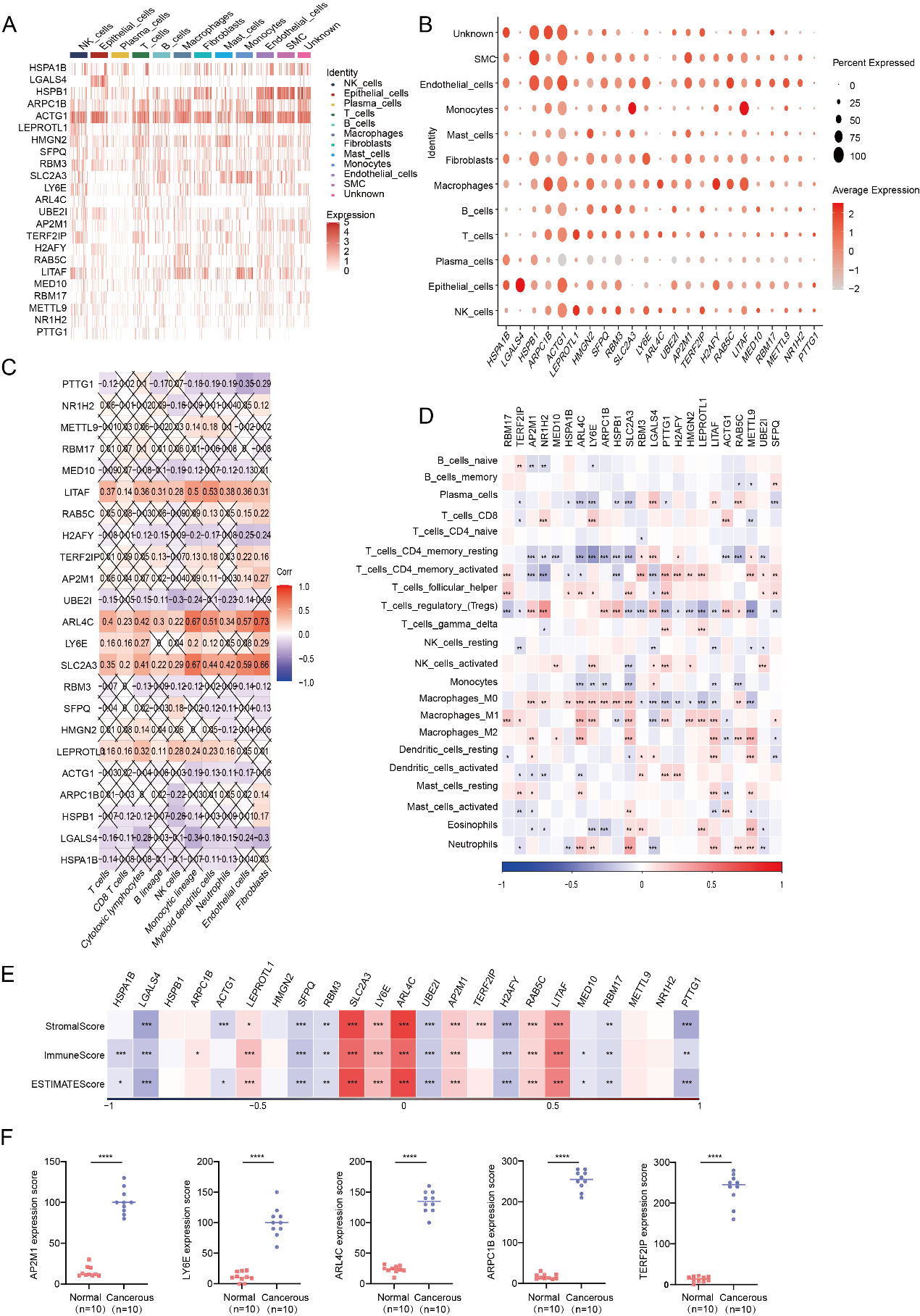


**Figure S3. Specific locations and functions of 23 core genes.**

A-B. Gene expression of 23 model genes in single cell transcriptome

C-D. The association analysis of 23 model genes with immune cells

E. Estimate score of 23 model genes

F. Statistical analysis of IHC results of AP2M1, LY6E, ARL4C, ARPC1B, TERF2IP. Statistical analysis was performed using the Mann-Whitney test. Statistical significance: *p < 0.05, **p < 0.01, ***p < 0.001, ****p < 0.0001.


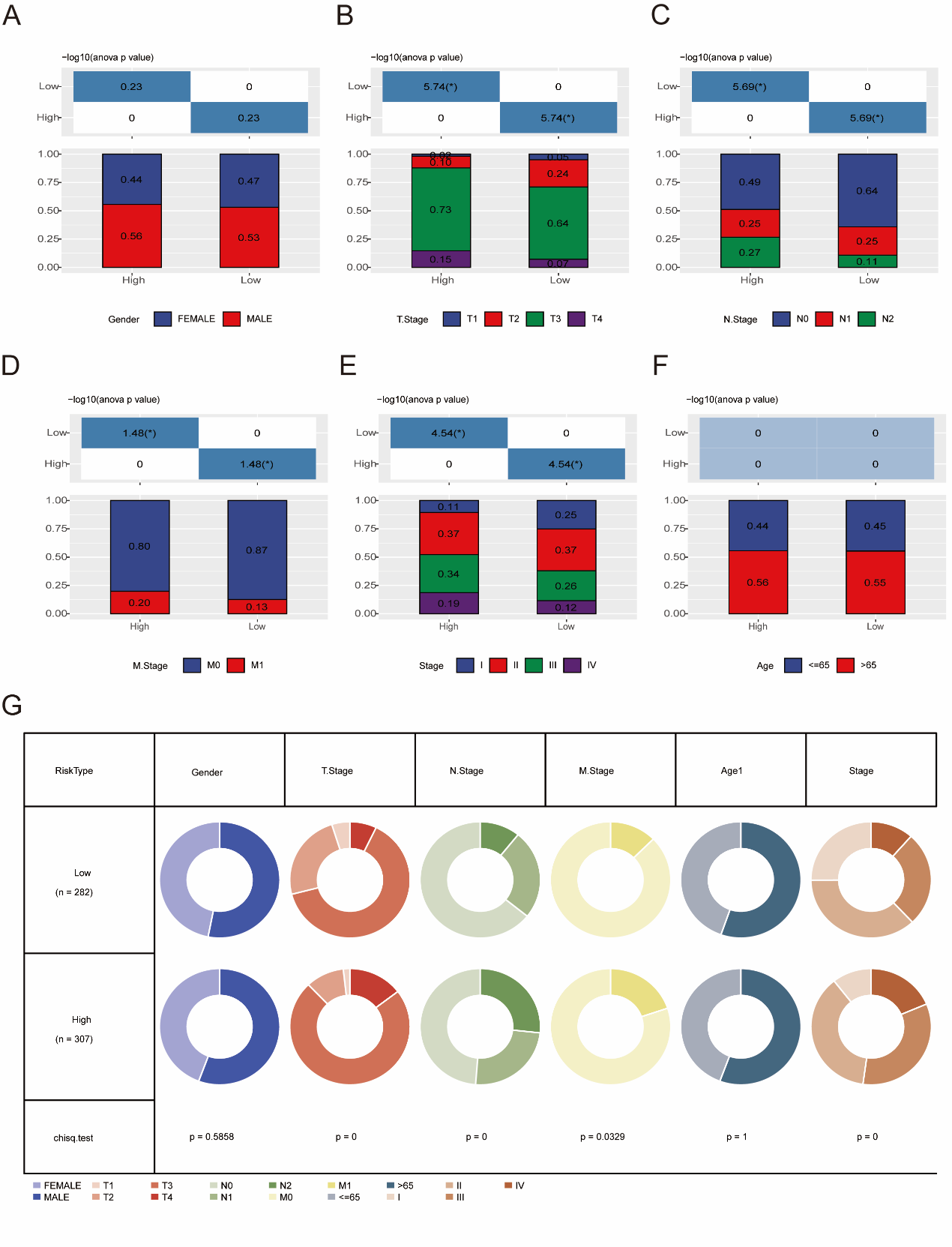


**Figure S4.** **Prognostic value of the LRGS risk.**

A-F. Lineplot Depicting the Relationship Between Different Clinical Factors and LRG Risk Score (A. Gender, B. T Stage, C. N Stage, D. M Stage, E. Overall Stage, F. Age)

G. Circus Plot Representing Different Clinical Factors in Two Signature Score Groups.

**Captions for Additional file Tables 1 to 16**

**Table S1.** Lactylation related genes from previous research.

**Table S2.** Top 10 marker genes in 22 clusters.

**Table S3.** Marker gene reference for all cells.

**Table S4.** Marker gene reference for immune cells.

**Table S5.** DEGs identified from high lactylation and low lactylation group.

**Table S6.** Top 100 Lactylation-Associated Genes (CORGs).

**Table S7.** Lactylation-related-genes indentified from our study.

**Table S8.** GSVA analysis between high lactylation and low lactylation group.

**Table S9.** GO pathways enriched in high lactylayion cells.

**Table S10.** DO pathways enriched in high lactylayion cells.

**Table S11.** Construction of the LRGS model.

**Table S12.** GO pathways enriched in 23 model genes.

**Table S13.** GSVA analysis between high risk and low risk group.

**Table S14.** Specific function of drugs screened from CTRP and PRISM.

**Table S15.** Association between core genes and specific drugs.

**Table S16.** Primers used in this study.
